# A transcriptional and functional analysis of heat hardening in two invasive fruit fly species, *Bactrocera dorsalis* and *Bactrocera correcta*

**DOI:** 10.1111/eva.12793

**Published:** 2019-04-10

**Authors:** Xinyue Gu, Yan Zhao, Yun Su, Jiajiao Wu, Ziya Wang, Juntao Hu, Lijun Liu, Zihua Zhao, Ary A. Hoffmann, Bing Chen, Zhihong Li

**Affiliations:** ^1^ Department of Entomology, College of Plant Protection China Agricultural University Beijing China; ^2^ Guangdong Inspection and Quarantine Technology Center Guangzhou China; ^3^ Redpath Museum McGill University Montreal Quebec Canada; ^4^ Department of Biology McGill University Montreal Quebec Canada; ^5^ School of BioSciences, Bio21 Institute University of Melbourne Parkville Victoria Australia; ^6^ State Key Laboratory of Integrated Management of Pest Insects and Rodents, Institute of Zoology Chinese Academy of Sciences Beijing China; ^7^ Present address: College of Life Sciences Hebei University Baoding China

**Keywords:** expression plasticity, hardening response, *Hsp23*, invasive species, thermal adaptation

## Abstract

Many insects have the capacity to increase their resistance to high temperatures by undergoing heat hardening at nonlethal temperatures. Although this response is well established, its molecular underpinnings have only been investigated in a few species where it seems to relate at least partly to the expression of heat shock protein (*Hsp*) genes. Here, we studied the mechanism of hardening and associated transcription responses in larvae of two invasive fruit fly species in China, *Bactrocera dorsalis* and *Bactrocera correcta*. Both species showed hardening which increased resistance to 45°C, although the more widespread *B. dorsalis* hardened better at higher temperatures compared to *B. correcta* which hardened better at lower temperatures. Transcriptional analyses highlighted expression changes in a number of genes representing different biochemical pathways, but these changes and pathways were inconsistent between the two species. Overall *B. dorsalis* showed expression changes in more genes than *B. correcta*. *Hsp* genes tended to be upregulated at a hardening temperature of 38°C in both species, while at 35°C many *Hsp* genes tended to be upregulated in *B. correcta* but not *B. dorsalis*. One candidate gene (the small heat shock protein gene, *Hsp23*) with a particularly high level of upregulation was investigated functionally using RNA interference (RNAi). We found that RNAi may be more efficient in *B. dorsalis*, in which suppression of the expression of this gene removed the hardening response, whereas in *B. correcta* RNAi did not decrease the hardening response. The different patterns of gene expression in these two species at the two hardening temperatures highlight the diverse mechanisms underlying hardening even in closely related species. These results may provide target genes for future control efforts against such pest species.

## 1 INTRODUCTION

During the invasive and adaptive process, species often encounter novel environmental conditions that require adaptation through genetically based evolutionary changes, phenotypic plasticity or a combination of these processes (Gibert et al., 2016; Vázquez, Gianoli, Morris, & Bozinovic, 2017). Phenotypic plasticity or evolutionary changes can help buffer organisms from environmental changes and thereby help their establishment and expansion, and even be the target of selection (Wellband & Heath, 2017). Many studies that consider the ability of plastic changes to buffer environmental effects consider temperature extremes, which play an important role in the success of the invasive process (David et al., 1997; Delpuech et al., 1995; Klepsatel et al., 2013).

Resistance to extreme high temperature represents a complex of traits that have been strongly affected by the environment experienced previously (Wos & Willi, 2018). Heat hardening is one component of resistance, involving the rapid induction of protective biochemical and physiological mechanisms, which markedly enhance heat resistance (Malmendal et al., 2006). This process of heat hardening in insects is regarded to be related to potential molecules, physiological changes or the differential expression of genes, such as the expression of heat shock protein (*Hsp*) genes (Borchel, Komisarczuk, Rebl, Goldammer, & Nilsen, 2018; Dahlgaard, Loeschcke, Michalak, & Justesen, 1998; Manjunatha, Rajesh, & Aparna, 2010; Sisodia & Singh, 2006; Sørensen, Kristensen, & Loeschcke, 2003; Willot, Gueydan, & Aron, 2017). The induction of genes such as *Hsp* varies with the intensity of thermal hardening stress and the insect's physiological state (King & MacRae, 2015). In *Drosophila,* hardening following a nonlethal heat stress activates a heat shock response through altering the transcription and translation of a set of genes including small *Hsp* genes (*sHsps*) and other *Hsps* including *Hsp70* (DiDomenico, Bugaisky, & Lindquist, 1982; Malmendal et al., 2006; Sørensen, Nielsen, Kruhøffer, Justesen, & Loeschcke, 2005). Other insects whose hardening responses have been characterized include the armyworm *Mythimna separata,* where preheating larvae enhances expression of genes encoding superoxide dismutase 1, catalase and *Hsp70*, the whitefly *Bemisia tabaci*, where *Hsp23, 70* and *90* are upregulated, and the ant *Cataglyphis mauritanica,* where hardening is associated with the expression of two *Hsc* *70‐4* cognates (Díaz, Orobio, Chavarriaga, & Toro‐Perea, 2015; Matsumura, Matsumoto, & Hayakawa, 2017; Willot et al., 2017).

**Figure 1:**
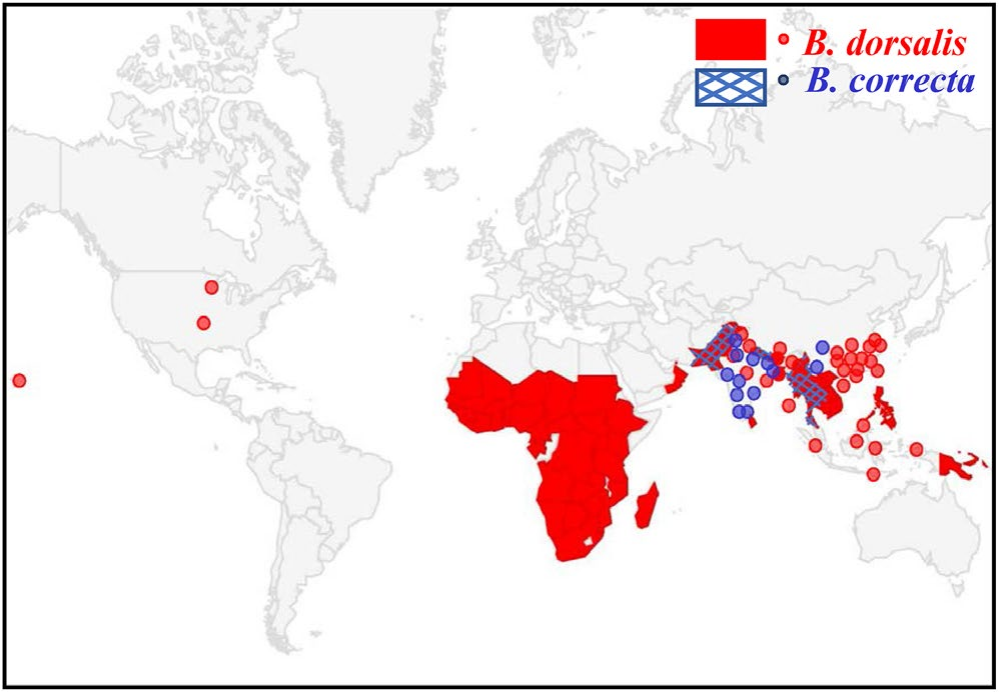
Global distribution map of *Bactrocera correcta* and *B. dorsalis.* The global distribution of the two species based on the information from GBIF (https://www.gbif.org/) and CABI (https://www.cabi.org/). The blue dots and areas represent the distribution of *B. correcta,* and the red dots and areas represent the distribution of *B. dorsalis*

*Bactrocera dorsalis* (Hendel) and *B. correcta* (Bezzi) (Diptera: Tephritidae) are invasive pests that damage fruits and vegetables (Permpoon, Aketarawong, & Thanaphum, 2011). These species have been investigated because of their increasingly wide distributions and repeated invasions. The *Bactrocera* genus has a competitive advantage for oviposition over other fruit flies such as *Ceratitis capitata* which is one of the most devastating and invasive worldwide pests (Liu, Zhang, Hou, Ou‐Yang, & Ma, 2017; Malacrida et al., 2007). The two *Bactrocera* species are similar in many biological attributes such as mating duration, oviposition preference, and offspring performance as well as sharing a common origin. However, the geographical distribution of *B. dorsalis* is now much wider than that of *B. correcta* both in China and elsewhere in the invasive range, which *B. dorsalis* has invaded including Hawaii, Kenya, and Tahiti and gradually displaced pre‐established *Ceratitis* species in recent years (Figure 1; Hu, Chen, & Li, 2014; Liu et al., 2014; Liu, Jin, & Ye, 2013; Lux, Copeland, White, Manrakhan, & Billah, 2003; Reitz & Trumble, 2002). Thus far, there are no records of *B. dorsalis* being displaced by other tephritid fly species (Liu et al., 2017).

Temperature tolerance may be one of the key factors influencing the distribution of Tephritidae like *B. dorsalis* and *B. correcta* (Hu et al., 2014; Liu & Ye, 2009; Pieterse, Terblanche, & Addison, 2017; Qin, Ni, et al., 2015; Qin, Paini, Wang, Fang, & Li, 2015). *B. dorsalis* and *B. correcta* have similar cold tolerance to *C. capitata* that is known to be capable of adapting to a wide range of climates; however, *B. correcta* is more susceptible to heat than *B. dorsalis* (Hallman, Myers, El‐Wakkad, Tadrous, & Jessup, 2013; Hu et al., 2014; Myers, Cancio‐Martinez, Hallman, Fontenot, & Vreysen, 2016; Papadopoulos, 2008). This may reflect species differences in thermal tolerance, mediated through transcription changes involving genes such as *Hsp70* and *Hsp90* (Hu et al., 2014). For instance in *Liriomyza, Hsp* gene expression at different temperatures in two *Liriomyza* likely influenced their geographical distribution (Huang & Kang, 2007; King & MacRae, 2015).

However, mechanisms that underpin heat hardening in *Bactrocera* species and how they might contribute to differences among the species are poorly characterized. As in other insects, there are limited data on how species might differ in gene transcription under hardening and how any differences relate to temperature adaptation and organism performance (Clarke, 2003). In this paper, we considered whether differences in temperature adaptability associated with hardening are linked to the regulation of certain genes or pathways, which in turn has contributed to the different distribution and invasive potential of the two species. We undertook a transcriptional analysis to identify mechanisms underlying differences of heat hardening responses in *B. dorsalis* and *B. correcta*. We first characterized the survival of these species under heat stress after a series of hardening treatments. Then, we undertook a transcriptional analysis to identify key pathways and genes involved in hardening. Lastly, quantitative real‐time PCR (qRT‐PCR) and RNA interference (RNAi) were used in a functional analysis of these two species around one gene that seemed particularly important in the hardening response and contributed to the different hardening response of these species.

## 2 MATERIALS AND METHODS

### 2.1 Samples

*Bactrocera dorsalis* and *B. correcta* were sourced from their first invaded range in China, from Guangdong province (N 23.40, E 113.22) for *B. dorsalis* with the annual mean temperature 21.7°C and from Yunnan province (N 23.60, E 102) for *B. correcta* with the annual mean temperature 25.8°C (Li, Wu, Chen, Wu, & Li, 2012; Liu & Ye, 2009). Cultures of these species were maintained in the laboratory for approximately 10 generations, across 3 years, at a temperature of 25°C, a humidity of 70% and light period of 10D:14L in an environment‐controlled incubator (Guo, Zhao, Liu, Li, & Shen, 2018; Nyamukondiwa, Terblanche, Marshall, & Sinclair, 2011; Weldon, Nyamukondiwa, Karsten, Chown, & Terblanche, 2018). Cultures were maintained by turning over flies across three cages (each 45 cm × 45 cm × 50 cm), with 200 individuals per cage. To reduce the risk of commonly observed inbreeding in fruit flies, we also regularly initiated new cultures of the species from the same provinces as above and added 30 new field fruit flies per cage from these cultures every half year (Hoffmann & Ross, 2018). Adult flies were given 25% sucrose and 75% peptone as their diet and immature stages were cultured on an artificial diet described by Yuan et al. (2006). The most temperature‐sensitive development stage (7‐day‐old 3rd early‐instar larvae) of these two species was chosen for hardening experiments (Hu et al., 2014; Jang, 1991). The 3‐day‐old 1st instar larvae for hardening response study and 4‐day‐old 2nd instar larvae for heat tolerance study of *B. dorsalis* and *B. correcta* were used in dsRNA‐feeding experiments.

### 2.2 Survival analysis under hardening

Five replicates with 30 larvae of each species, *B. dorsalis* and *B. correcta,* were exposed to heat hardening temperatures ranging from 34 to 40°C as described in Hu et al. (2014) for 4 hr in a circulating water bath (PolyScience Programmable Temperature Controller, Total Temperature Instrumentation, Inc., USA), while the control group was maintained at 25°C. After pretreatment, all tubes were returned to 25°C for 1 hr before exposure to heat stress at 45°C for 1 hr in the water bath, which allowed various heat shock proteins and other genes to develop (Hoffmann, Sørensen, & Loeschcke, 2003; Sørensen & Loeschcke, 2001). We chose 45°C for 1 hr as an extreme thermal stress which caused mortality in the range 40%–50% without heat hardening (Hu et al., 2014; Nyamukondiwa et al., 2011). After exposure to the heat stress, the larvae were returned to 25°C and their survival rate was scored after 4 hr (Supporting Information Figure S1A). During the thermal treatments, samples were enclosed in 2 ml tubes with a hole in the lid, topped with 4 g diet to ensure the food supply and humidity. Tubes were suspended in a circulating water bath set to the desired temperature. The control 25°C flies were also handled in the same way as the flies in the hardening experiments. Each replicate had 30 larvae in the 2 ml tube and each hardening treatment involved five biological replicates.

### 2.3 RNA extraction and cDNA synthesis

Individuals for mRNA sequencing, transcriptome verification and the detection of RNA interference efficiency in each species were collected randomly for RNA extraction. RNA was extracted from whole body of third instar larvae using an RNA simple Total RNA kit (Tiangen, China). cDNA was synthesized from 1,000 ng total RNA using PrimeScript™ RT reagent kit with gDNA Eraser (Perfect Real Time; Takara, Japan) following the manufacturer's instructions.

### 2.4 Transcriptome and transcriptome analysis of heat hardening

For mRNA sequencing, total RNA (5 μg) was isolated from three groups of 30 3rd early‐instar larvae after exposing them to specific temperatures (25, 35 and 38°C) for 4 hr followed by 25°C for 1 hr for both *B. dorsalis* and *B. correcta* (Supporting Information Figure S1B) using an RNA simple Total RNA kit as described in Section 2.3. The quantity and integrity of the RNA were assessed with a Qubit^®^ RNA Assay kit in Qubit^®^2.0 Fluorometer (Life Technologies, CA, USA) and an RNA Nano 6000 Assay kit of the Agilent Bioanalyzer 2100 system (Agilent Technologies, CA, USA). For use as a template, mRNA was enriched with a NEBNext Poly (A) mRNA Magnetic Isolation Module (NEB, E7490, Ipswich, MA, USA). RNA was chemically fragmented into 200–700 nt fragments and converted into single‐stranded cDNA using random hexamer priming, followed by second‐strand cDNA synthesis using DNA Polymerase I and RNase H. The purified double‐stranded cDNA products were processed via magnetic beads, end repaired and ligated to adaptors. All libraries were prepared using NEBNext^®^Ultra™ RNA Library Prep kit for Illumina^®^ (NEB, USA) according to the manufacturer's protocol. Finally, after the assessment of fragment sizes through 2% agarose gel electrophoresis and validation by quantitative real‐time PCR using a library quantification kit/Illumina GA Universal (KAPA, Wilmington, MA, USA), all libraries were sequenced on an Illumina HiSeq^TM^ 2000 instrument (Illumina) at the Biomarker Technologies company (Beijing, China). The clustering of index‐coded samples was performed on a cBot Cluster Generation System using TruSeq PE Cluster kit v3‐cBot‐HS (Illumina, San Diego, CA, USA), and paired‐end reads were generated through sequencing.

To remove adapter contamination, low‐quality bases and bases artificially introduced during library construction, we trimmed all raw reads using Trimmomatic 0.32 (http://www.usadellab.org/cms/index.php?page=trimmomatic) with the following parameters: Phred33 LEADING: 3 TRAILING: 3 SLIDING WINDOW: 1:10 MINLEN: 75 before transcript assembly, while the unpaired reads were discarded (Bolger, Lohse, & Usadel, 2014). The clean reads were mapped to the sequences in the rRNA database of all published insects downloaded from NCBI to discard rRNAs using SOAP (Li, Li, Kristiansen, & Wang, 2008). Only the clean reads with the standard of Q30 > 85% and processed with Trimmomatic 0.32 and SOAP were used for further analysis. As we wanted to compare the two species and only the genome for *B. dorsalis* was available (ASM78921v2; https://i5k.nal.usda.gov/Bactrocera_dorsalis), Trinity for research without the need for a genome sequence with set parameters (min_kmer_cov: 2 min_contig_length: 200 group_pairs_distance: 500) was used for de novo transcriptome assembly to obtain corresponding transcripts (Guo et al., 2018; Haas et al., 2013). The two species were firstly assembled to get their own UniGene database, and then the general UniGene library was obtained by clustering the two individual databases through CD‐Hit to compare the expression and analyze differences between the two species. Trinity was used to break down clean reads into short fragments (Grabherr et al., 2011). Reads of certain lengths of overlap were combined in contigs to form longer fragments. These assembled unigenes were further processed for sequence splicing and redundancy removal using sequence clustering software to acquire maximum length nonredundant unigenes (Fu, Niu, Zhu, Wu, & Li, 2012). Briefly, Trinity was used for assembly to obtain corresponding transcripts, and we retained the transcript with the highest read coverage and removed the transcript with the lowest read coverage for each subcomponent. The transcripts selected in the clustering united as unigenes using the De Bruijn graph algorithm and CD‐HIT to reduce sequence redundancy and improve the performance of other sequence analyses (Fu et al., 2012; Yang & Smith, 2013).

For functional annotation, sequence alignments of unigenes to the protein databases NR (nonredundant RefSeq proteins—NCBI), Swiss‐Prot, GO (gene ontology), COG (Clusters of Orthologous Groups), KOG (euKaryotic Orthologous Groups), eggNOG4.5 and KEGG (Kyoto Encyclopedia of Genes and Genomes) were undertaken by using BLAST (*E*‐value < 1e‐5) (Altschul et al., 1997; Ashburner et al., 2000; Bairoch et al., 2005; Deng et al., 2006; Huerta‐Cepas et al., 2015; Kanehisa, Goto, Kawashima, Okuno, & Hattori, 2004; Tatusov, Galperin, Natale, & Koonin, 2000). After predicting the amino acid sequence of unigenes through comparisons with the Pfam database, HMMER software was used to obtain annotation of protein function (*E*‐value < 1e‐10) (Dahlgaard et al., 1998; Finn et al., 2013). Bowtie was used to align the reads to the Trinity transcripts and estimate the number of RNA‐Seq fragments (Langmead, Trapnell, Pop, & Salzberg, 2009). With Bowtie alignment, FPKM (fragments per kilobase of exon per million reads mapped) were quantified using RSEM software (Li & Dewey, 2011). To evaluate the correlation of gene expression across different replicates, we calculated Pearson's correlation coefficient (*r*) between the three biological replicates (Schulze, Kanwar, Gölzenleuchter, Therneau, & Beutler, 2012). The analysis of differentially expressed genes (DEGs) was conducted in three steps. Firstly, read counts were adjusted by DESeq package through scaling normalized factor for each sequenced library before analysis (Anders & Huber, 2010; Wang, Feng, Wang, Wang, & Zhang, 2009). Secondly, differential expression analysis of samples was performed using the DESeq (Anders & Huber, 2010; Wang et al., 2009). In this analysis, *p*‐value adjusted by the Benjamini–Hochberg approach was used to decrease false positives (Ferreira & Zwinderman, 2006). Lastly, the standard fold change>|2| was set as the threshold for differential expression. To visualize transcription differences among treatments, a principal component analysis (PCA) was performed in R (2. 22. 0) with the *prcomp* function. This was done with all the FPKM of DEG of three replicates in *B. correcta* (Bc) and *B. dorsalis* (Bd) under three temperatures. Heat maps of absolute DEG expression were generated as logarithmic values that normalized FPKM for hardening temperatures (HT) by dividing by FPKM for control temperature (CK) (log_10_^DEG_FPKM of HT/DEG_FPKM of CK^). GO enrichment analysis was implemented through the *topGO* R packages based a Kolmogorov–Smirnov test (Alexa & Rahnenfuhrer, 2010). KOBAS software was used to test statistically significant enrichment of differentially expressed genes in KEGG pathways (Xie et al., 2011).

### 2.5 Quantitative Real‐time PCR for transcriptome verification and the detection of RNA interference efficiency

In order to validate the results from the transcriptome analysis, we quantified the expression profiles of candidate genes *Hsp23*, *Hsp70* and *Hsp90* for *B. dorsalis* and *B. correcta*. Thirty individuals for transcriptome verification (two species with three temperature treatments: 25, 35 and 38°C for 4 hr followed by 25°C for 1 hr as described in Supporting Information Figure S1B) were collected randomly. For the detection of RNA interference efficiency, 10 individuals fed on dsRNA after 96 hr in each species were randomly selected for *Hsp23* expression detection. RNA extraction and cDNA synthesis followed the description in Section 2.3. The RNAi efficiency was quantified by quantitative real‐time PCR using SYBR^®^ Premix Ex Taq™ II (TliRNaseH Plus; Takara, Japan) on an ABI 7500 instrument (Applied Biosystems Europe, Belgium). All RNA samples were performed with same methods and analyzed in triplicate. The reaction included 1 μl cDNA, 12.5 μl SYBR Green mix, 1 μl each of forward and reverse primers (10 p.m.), 0.5 μl ROX Reference Dye II and 9 μl ddH_2_O. The thermocycler conditions were 95°C for 30 s, followed by 40 cycles at 95°C for 5 s and 60°C for 34 s. Melting curve analysis was performed at the end of each expression analysis, using the following conditions: 95°C for 15 s, followed by 60°C for 60 s with the decreasing rate of 1°C/s from 95°C. All the primers used are described in Supporting Information Table S4, and the amplification efficiency of all the primers in these two species is close to 100% (Supporting Information Table S5; Hu et al., 2014; Shen, Huang, Jiang, Dou, & Wang, 2013). We evaluated the performance of five reference genes, including *18s ribosomal RNA (18s), ribosomal protein L13 (RPL13), succinate dehydrogenase *(*SD*),* α‐tubulin (α‐TUB)* and *β‐tubulin (β‐TUB)* in *B. dorsalis* and *B. correcta* using three software‐based approaches (BestKeeper, NormFinder and geNorm) and *18s* was chosen as the reference gene in the experiment (Supporting Information Table S6). The qRT‐PCR data were analyzed using the 2^−ΔCT^ method (Chen & Wagner, 2012). Five biological replicates were carried out for statistical analysis. All results from experimental replicates were analyzed with Student's *t* tests or one‐way analyses of variance (ANOVAs) with SPSS 20 (IBM Corporation, USA).

### 2.6 Cloning the open reading frame of *Hsp23*

*Hsp23* was selected as a candidate hardening gene based on the analysis of the transcriptomic data of *B. dorsalis* and *B. correcta*. To verify the open reading frame (ORF) of *Hsp23* in each species, due to the high similarity on sequence, one pair of primers named *Hsp23*‐dsRNA‐F/R (Supporting Information Table S4) was designed using Primer Premier 5.0 software (PREMIER Biosoft International, USA) based on the sequence information from transcriptome. The ORF and conserved domain were identified with the ORF Finder software (http://www.ncbi.nlm.nih.gov/gorf/gorf.html).

### 2.7 Phylogenetic analysis

The integrity of homologous amino acid sequences of other species was retrieved from the NCBI server (https://www.ncbi.nlm.nih.gov/; Dou et al., 2017). Sequences were first aligned by the conserved sequences, and then, phylogenetic analysis was performed using the neighbor‐joining method in the Molecular Evolutionary Genetics Analysis software (MEGA version 5.1; Tamura et al., 2011). All the positions that contained gaps and missing data were eliminated before alignment and phylogenetic analysis.

### 2.8 Validation of *Hsp23* role through dsRNA

*Hsp23* was chosen for functional analyses because it is the most expressed gene in *B. dorsalis*, the species with the stronger invasion potential, which likely involved the ability of this species to adapt to increased temperature. While *Hsp70* was the most expressed gene in *B. correcta,* this gene was not selected for further analysis given that it did not stand out in terms of expression changes in *B. dorsalis*, whereas *Hsp23* was upregulated in both species (Figure 7).

Double‐stranded RNA of *Hsp23* (ds*Hsp23*) was used to knock down the expression of the target gene of *B. dorsalis* and *B. correcta*. Green fluorescent protein (ds*GFP*) was used as a negative control. The dsRNA was synthesized by the primers named *Hsp23*‐dsRNA‐F/R and Hsp23‐F/R‐T7 in Supporting Information Table S4 and using the T7 RiboMAX Express RNAi system (Promega). For larval feeding, three grams artificial diet with 30 μl of a dsRNA solution (1,000 ng/μl) was put into a 50 ml tube. Forty 3‐day‐old 1st instar larvae for hardening response study or 4‐day‐old 2nd instar larvae for heat tolerance study of *B. dorsalis* and *B. correcta* were collected and moved into a 50 ml tube with artificial diet, which had three holes in its lid for air. Five replicates per treatment were carried out and each replicate contained 40 larvae. For the temperature study, larvae were fed with ds*Hsp23* and ds*GFP* for 48 hr and transferred to new artificial diet for another 48 hr. After 96 hr feeding, the larvae developed to 3rd early‐ or late‐instar larvae.

### 2.9 Extreme heat stress and hardening response after feeding ds*Hsp23*

After 96 hr dsRNA feeding, 10 larvae of each species of *B. dorsalis* and *B. correcta* were killed for detecting *Hsp23* gene expression. The rest of the 3rd late‐instar larvae were transferred to one 2 ml tube with diet for high temperature exposure. We chose 3rd late‐instar larvae because of their high survival rate under extreme temperature to study the function of *Hsp23* on heat tolerance (Jang, 1991). The larvae were directly exposed to heat stress at 45°C for 1 hr followed by 4 hr at room temperature before scoring. As there was no response in the *Hsp23*‐knocking down in *B. correcta* at extreme high temperature, hardening response was only studied in *B. dorsalis*. Five groups of 40 3rd early‐instar larvae with low survival rate under extreme temperature were used to study the function of *Hsp23* on heat hardening, which showed the recovery of *Hsp23* expression could increase survival rate. After the feeding, the larvae were exposed to heat hardening (35 and 38°C) and control (25°C) temperatures separately for 4 hr and returned to 25°C for 1 hr (Supporting Information Figure S1B). Ten of the flies were frozen in liquid nitrogen for the detection of *Hsp23* expression. The other 30 flies were exposed to heat stress at 45°C for 1 hr in a water bath (Supporting Information Figure S1A). After heat stress, the larvae were moved back to 25°C and their survival rate scored after 4 hr. Each treatment was replicated five times.

## 3 RESULTS

### 3.1 Hardening response of *B. dorsalis* and *B. correcta*

**Figure 2:**
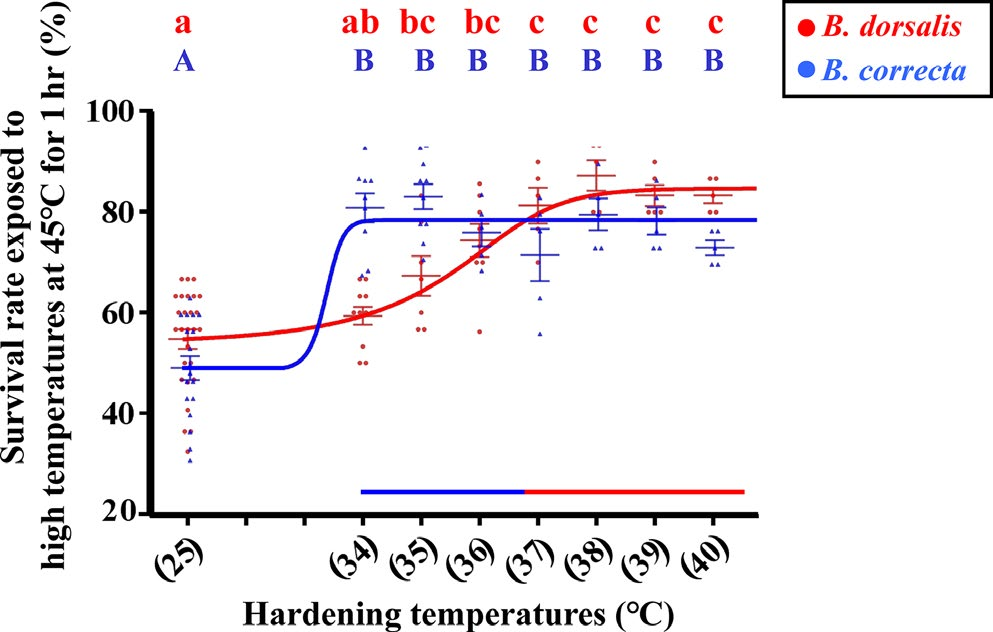
Heat hardening response of *Bactrocera correcta* and *B. dorsalis.* The survival rate of 7‐day‐old 3rd instar larvae exposed to high temperature after heat hardening treatments. The temperatures used for hardening are indicated on the *x*‐axis. The blue line represents the fitted curve of larval survival rate using an asymmetric model for* B. correcta.* The red line shows the fitted curve for *B. dorsalis* using the same model*.* Different letters above the bars indicate significant differences at *p* < 0.05, as determined by a Tukey HSD test. The capital letters in blue refer to *B. correcta* and the lowercase letters in red refer to *B. dorsalis*. The error bars indicate 1 *SE*

We calculated the survival rate of 3rd instar larvae in the two species (Figure 2). Both species responded to the hardening treatments from 34 to 40°C, with increased survival rate under heat stress of varying degrees. However, species had different hardening responses. *Bactrocera dorsalis* performed better than *B. correcta* in the 37–40°C hardening range, while *B. correcta* differed from *B. dorsalis* in the 34–36°C range. A hardening temperature of 35°C produced the largest benefit for *B. correcta,* while 38°C led to the largest hardening response for *B. dorsalis*.

### 3.2 Transcriptome and bioinformatic analysis of *B. dorsalis* and *B. correcta*

**Figure 3:**
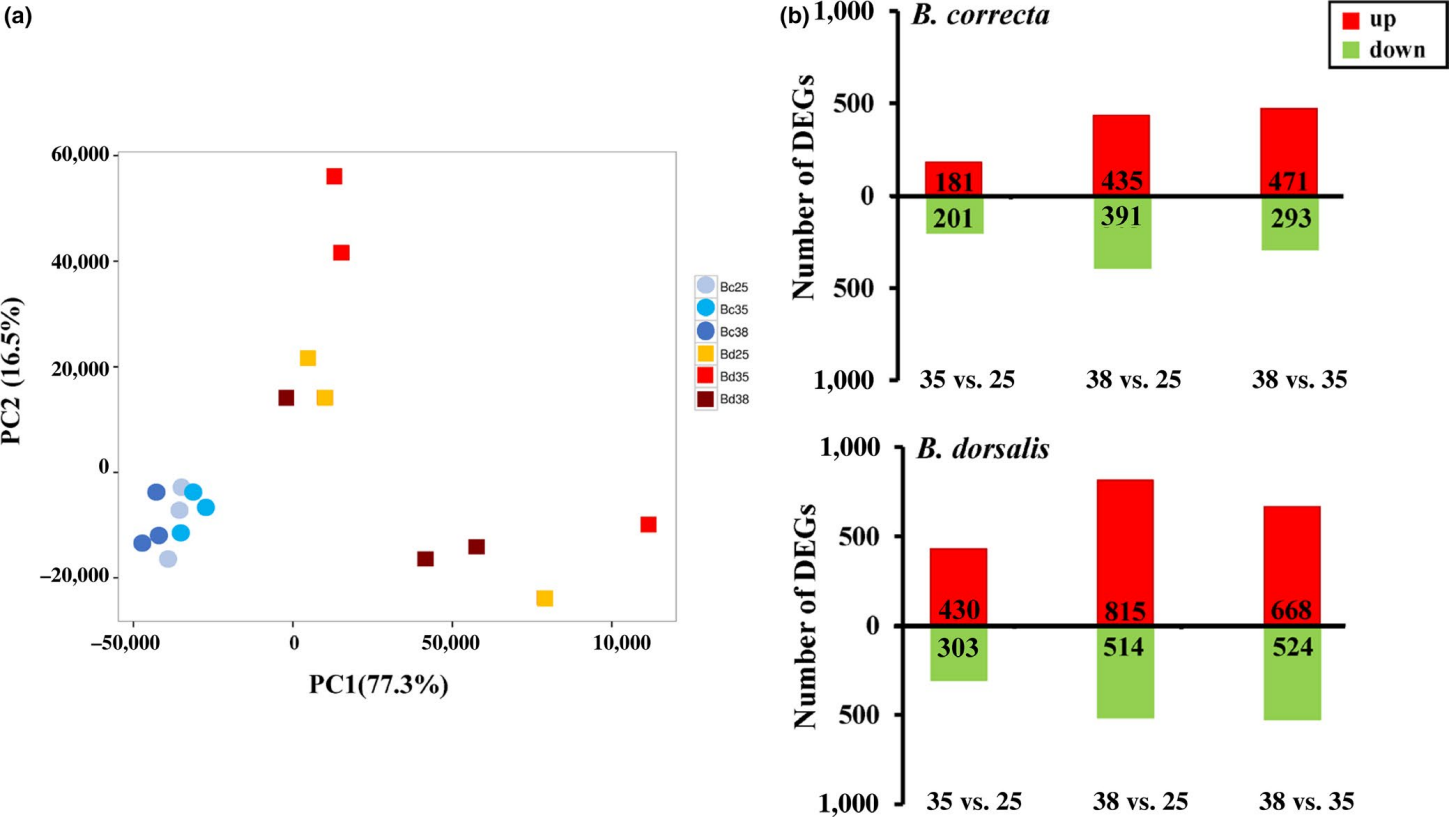
Transcriptome analysis of *Bactrocera correcta* (Bc) and *B. dorsalis* (Bd) after exposure to different temperatures*.* (a) Clustering of control and heat hardening groups based on their transcriptome profiles by principal component analysis (PCA) across both species. Specific colors and points represent replicates for hardening treatments and species as shown in the legend. PCA was computed in R using the princomp function and a correlation matrix with the expression data of all the genes. Whereas *B. correcta* treatments formed a tight group, *B. dorsalis* was more variable depending on temperature. (b) Number of differentially expressed genes (DEGs) with fold change >2 for *B. correcta* and *B. dorsalis.* The chart above represents the number of DEGs at 35 versus 25°C, 38 versus 25°C and 38 versus 35°C in *B. correcta,* and the chart below represents the number of DEGs at 35 versus 25°C, 38 versus 25°C and 38 versus 35°C in *B. dorsalis*. Up‐ (red) and downregulated (green) unigenes are quantified

**Figure 4:**
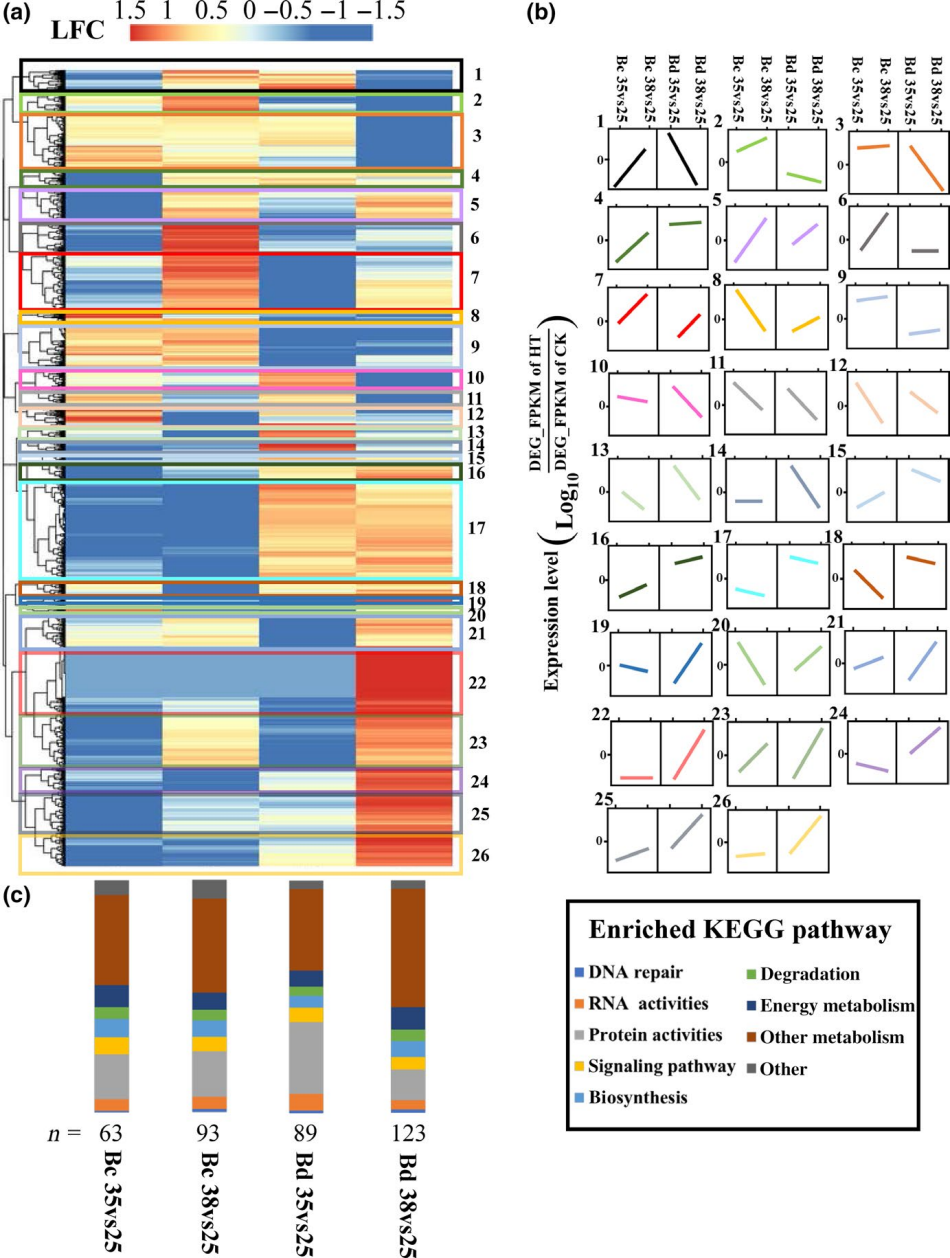
Heatmaps of DEGs under different hardening treatments (35, 38 and 25°C control) in *Bactrocera correcta* (Bc) and *B. dorsalis* (Bd)*.* (a) The heatmap of DEGs in *B. correcta* and *B. dorsalis.* Differential expression is shown as log10 fold changes (LFC) of hardening temperatures versus 25°C using the value of log_10_^FPKM for hardening temperatures/FPKM for control temperature^. For clarity, LFC values were capped at 1.5 and −1.5. The color bars besides the heatmaps indicate the relative size of the changes. (b) Differential expression patterns between the two species across treatments. (c) Classification of KEGG pathways under different hardening treatments. The number below the bars represents the number of enriched KEGG pathways for each experimental group

The percentage of bases with a Q30 score was over 92.64%, indicating that the sequencing was reliable. We obtained 94,821 unigenes from larval transcriptome sequencing, and a total of 42,931 unigenes were annotated in the larvae transcriptome from databases in these two species (Supporting Information Tables S1 and S2). The length distribution of the unigenes and statistics for larval unigenes assessment are shown in Supporting Information Figure S2 and Table S1, and the number of unigenes with a length >1,000 bp was 16,114. The mapped ratio for all the samples was over 76% (Supporting Information Table S3). In the transcriptome analysis, the PCA, bar charts and heatmaps of DGEs analyses revealed differences between the *Bactrocera* species under different hardening temperatures (Figures 3 and 4). In the PCA analysis of these two species, PC1 explained 77.3% of the variance in expression and clustered *B. dorsalis* separately from *B. correcta* (Figure 3a). Although PC2, which explained 16.5% of the variance, clustered treatment samples of *B. dorsalis* separately, they showed no clear separation of *B. correcta* treatments (Figure 3a). More genes changed expression levels in *B. dorsalis* (Figure 3b) and expression patterns differed between the species as well as hardening temperatures (Figure 4).

The variation in gene expression was analyzed in comparisons of 35 versus 25°C, 38 versus 25°C and 38 versus 35°C in the two species. Compared to *B. correcta*, we found that there were more genes differentially expressed in *B. dorsalis* under both hardening treatments (Figure 3b). For 35°C treatments, a total of 733 and 382 genes were differentially expressed, respectively, in *B. dorsalis* and *B. correcta* when compared to control treatments. In *B. dorsalis*, there were 430 upregulated genes and 303 downregulated genes (Figure 3b). There was relatively more upregulation in biological processing in cytochrome P450 of *B. dorsalis*, which reflected increasing P450 activities (Supporting Information Figures S3D and S4D). However, genes such as larval serum protein, dehydrogenase, cecropin and mucin were significantly downregulated. In *B. correcta*, 181 genes were upregulated and 201 genes were downregulated. Genes annotated with endocytosis, protein processing in endoplasmic reticulum and spliceosome in KEGG analysis were upregulated (Supporting Information Figures S3A and S4A). Downregulated genes were annotated with peroxisome, glycolysis, pyruvate metabolism and biosynthesis of amino acids (Supporting Information Figures S3A and S4A). These patterns indicated substantial changes in gene expression affecting a diversity of pathways.

In the comparison of 38 and 25°C, a total of 1,329 and 826 genes showed significantly different expression in *B. dorsalis* and *B. correcta,* respectively. In *B. dorsalis*, 815 genes were upregulated and 514 genes downregulated (Figure 3b). Genes related to lysosome, glycolysis, carbon metabolism and biosynthesis of amino acids were upregulated at 38°C (Supporting Information Figures S3E and S4E). Genes related to carbon metabolism, metabolism of xenobiotics by cytochrome P450, protein processing in endoplasmic reticulum and pentose and glucuronate interconversions were significantly downregulated (Supporting Information Figures S3E and S4E). These changes reflected energy metabolism such as glycolysis which may have provided energy for the hardening response, and protein processing in the endoplasmic reticulum which may affect the accumulation of unfolded proteins (Ron & Walter, 2007). In *B. correcta,* 435 genes were upregulated and 391 genes downregulated (Figure 3b). Genes related to endocytosis, protein processing in endoplasmic reticulum and spliceosome were upregulated at 38°C (Supporting Information Figures S3B and S4B), which was similar to the response at 35°C. Genes such as peptidoglycan‐recognition protein, cuticle protein and lambda‐crystallin homolog were significantly downregulated. For the KEGG annotation analysis, the regulated genes mainly involved the peroxisome, protein processing in endoplasmic reticulum and metabolism of xenobiotics by cytochrome P450 (Supporting Information Figures S3B and S4B), this suggests that heat stress may affect a range of oxidative processes.

When comparing the two hardening temperature conditions (35 vs. 38°C), 1,192 and 764 genes were detected with significantly different expression in *B. dorsalis* and *B. correcta,* respectively (Figure 3b). In contrast for *B. dorsalis*, a number of affected genes were involved in the biosynthesis of amino acids and carbon metabolism (Supporting Information Figures S3F and S4F). There were also many differentially expressed genes with unknown functions. In the KEGG analysis, most *B. correcta* genes related to the peroxisome, protein processing in endoplasmic reticulum and fatty acid activities (Supporting Information Figures S3C and S4C).

There were more genes and processes affected at 38°C than at 35°C (Figure 4a). Genes that showed certain expression patterns in the two species formed 26 clusters (Figure 4b). Genes in cluster 2 and 9 annotated with immune response such as defense response to bacterium and innate immune response, stress response and some monooxygenase activities were upregulated only in *B. correcta* at both hardening temperatures, while the related genes were downregulated in *B. dorsalis.* However, genes in cluster 13, 16, 17, 18, 20 and 24 related to many developmental processes were only upregulated in *B. dorsalis,* whereas genes in the same cluster 16, 17, 24 were downregulated in *B. correcta*. For the KEGG terms, enriched pathways numbered 89 in the 35 versus 25°C comparison and 123 in the 38 versus 25°C comparison for *B. dorsalis*, and the equivalent numbers for *B. correcta* were 63 (35 vs. 25°C) and 93 (38 vs. 25°C; Figure 4c).

**Figure 5:**
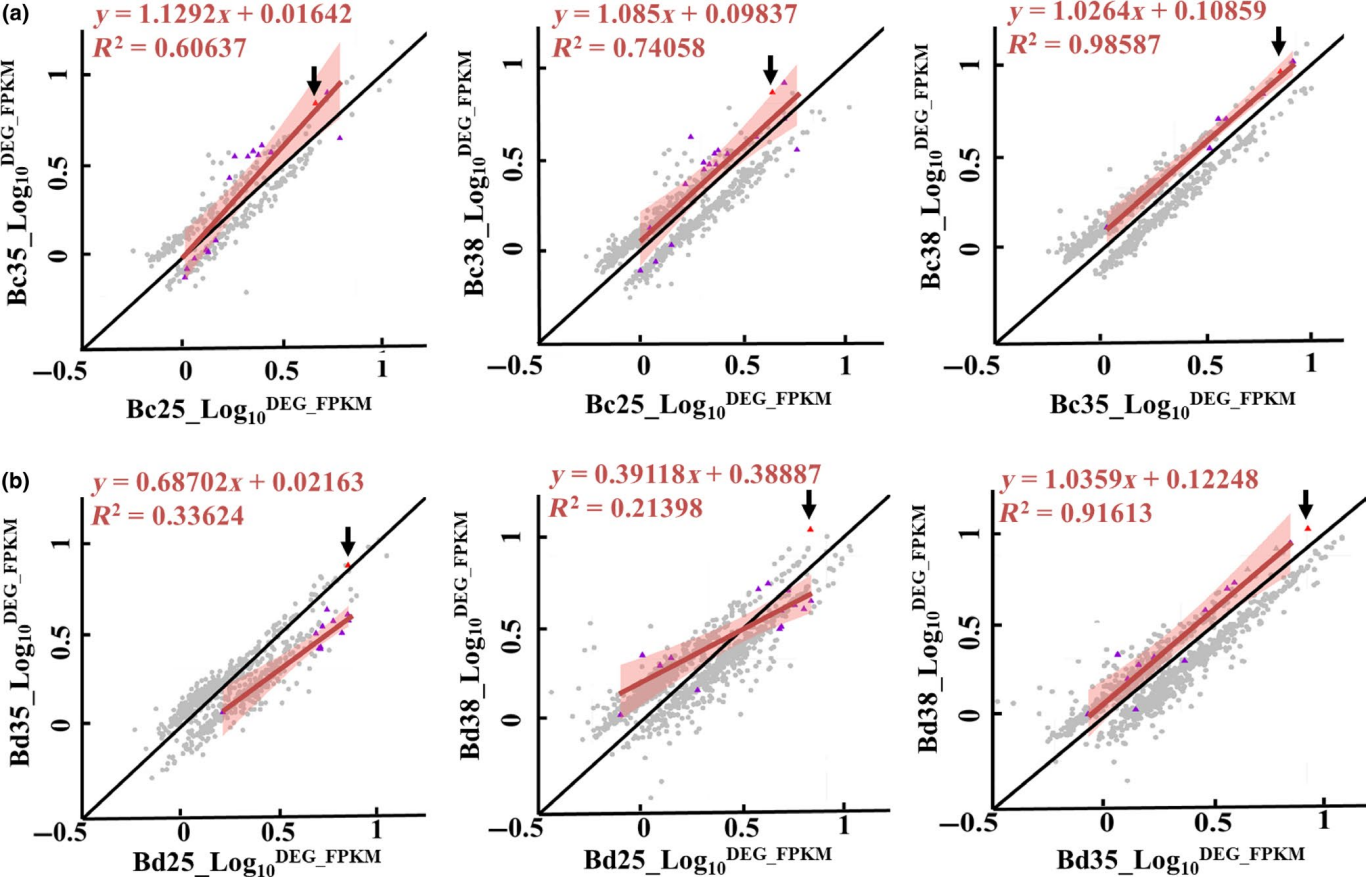
Relationship between mRNA levels for genes and heat shock protein genes (*Hsps*) with fold change more than 2 under hardening/control treatment comparisons (35, 38 and 25°C controls). DEGs are measured as a logarithmic value of mean FPKM for three replicates (log_10_^mean FPKM^) in *Bactrocera correcta* (Bc) and *B. dorsalis* (Bd). Transcripts encoding *Hsps* are shown as purple triangles, *Hsp23* is highlighted in red (arrow), and all other transcripts are represented by gray circles. The black line represents *k* = 1, and the red line and zone (±95% confidence bands) represent the least‐squares regression fit of *Hsp* transcripts, for which *Hsp23* was a significant outlier in *B. dorsalis* under the two hardening temperatures when compared to 25**°**C

Differentially expressed genes including up‐ and downregulated genes at different temperature treatments remained positively correlated in expression levels between treatments in the scatter plots (Figure 5). As *Hsps* were the largest family responding to stress among all the DEGs in these two species with the highest levels of expression (Figure 5) and *Hsp* genes such as *Hsp*70, *Hsp*90 and small heat shock protein (*sHsp*) have been reported as playing roles in high temperature responses previously (Borchel et al., 2018; Dahlgaard et al., 1998; Manjunatha et al., 2010; Sisodia & Singh, 2006; Sørensen et al., 2003; Willot et al., 2017), we considered *Hsps* separately. More *Hsp* genes were upregulated with higher expression levels in *B. correcta* than in *B. dorsalis,* except for *Hsp23*, which had a very high expression level compared to all other genes under different hardening conditions (Figure 5 and Supporting Information Figures S5 and S6 and Table S7). Also, many other *sHsp*s were associated with the hardening process, such as *Hsp18.4* and *Hsp20* (Supporting Information Figures S5 and S6 and Table S7)*.*

**Figure 6:**
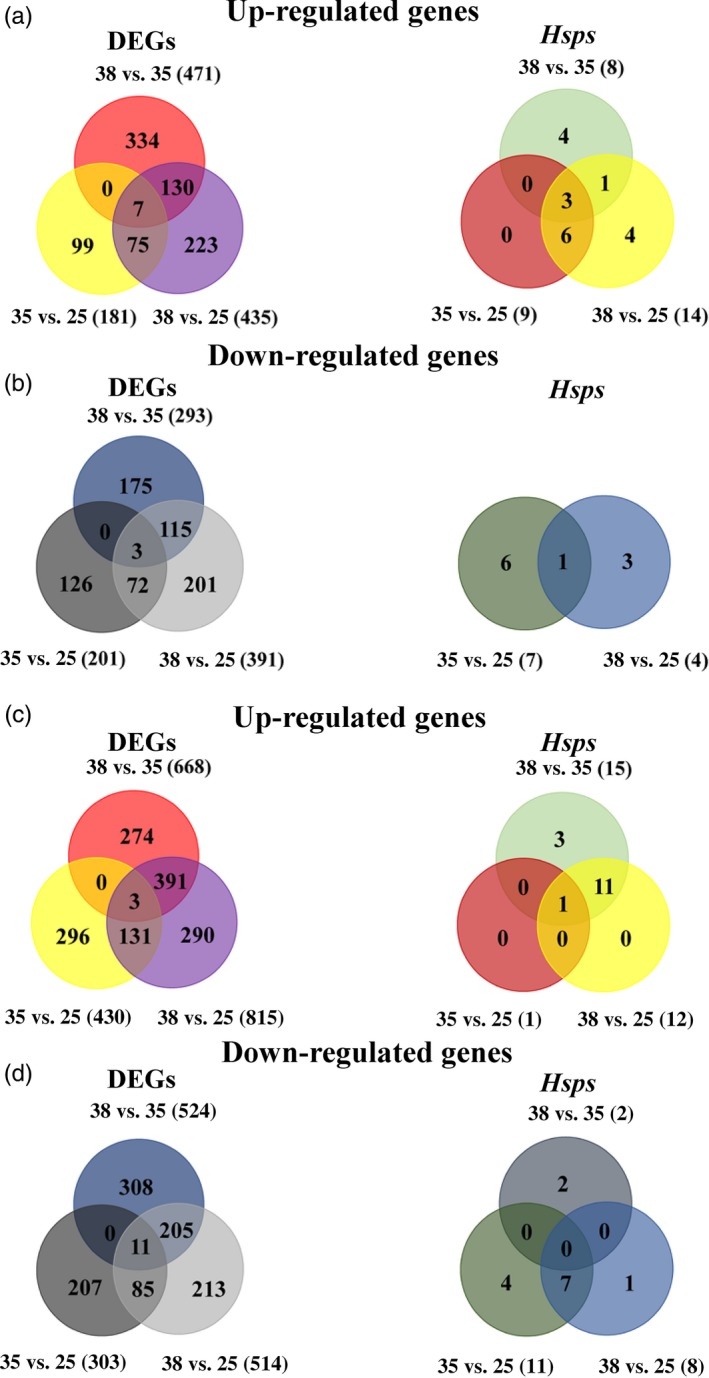
Wayne maps of up‐ (red, light green, yellow and purple) and downregulated (blue, dark green, light and dark gray) DEGs and *Hsp* genes that were differentially expressed (35 vs. 25℃, 38 vs. 25℃, 38 vs. 35℃) in *Bactrocera correcta* and *B. dorsalis.* (a) The upregulated DEGs and *Hsp* genes in *B. correcta.* (b) The downregulated DEGs and *Hsp* genes in *B. correcta.* (c) The upregulated DEGs and *Hsp* genes in *B. dorsalis.* (d) The downregulated DEGs and *Hsp* genes in *B. dorsalis.* Bold numbers in parentheses represent total numbers of regulated unigenes in these two species

Although there were common genes upregulated by both hardening treatments, *Hsp23* was the only annotated *Hsp* gene upregulated in all combinations of temperature groups in *B. dorsalis* through Wayne maps (Figure 6c)*.* The same *Hsp23* gene, among several *Hsp* genes, was also significantly upregulated in *B. correcta* (Figure 6a). Among all the genes upregulated under the two hardening temperatures (3 genes in *B. dorsalis* and 7 genes in *B.  correcta*), the *Hsp*s were the only BLAST genes identified as clear candidates. Some *Hsp* genes were downregulated, including *Hsp83,* which was downregulated when compared to the control groups under two hardening temperatures in these two species (Figure 6b,d and Supporting Information Table S7). In comparison with the other genes including the *Hsp* genes, the expression of *Hsp23* was at a high level at all temperatures (Figure 5) and associated with the weight of early pupae in *B. dorsalis* (Supporting Information Figure S7). In general, *Hsp23* is one of the highest expressed genes in all DEGs including *Hsps* in both species (Figure 5)*.*

### 3.3 Cloning and characterization of *Hsp23*

*Hsp23* from both species was cloned from cDNA. Based on the phylogenetic analysis and sequencing results, Bc*Hsp23* and Bd*Hsp23* were similar to each other, with a difference in nucleic acid and amino acid sequences of ORFs of only 17 out of 513 base pairs and 7 out of 170 amino acids.

### 3.4 Phylogenetic analysis of *sHsp* from different insect species

The NJ tree (Supporting Information Figure S8) showed two annotated *Hsp23* genes in *B. correcta* and *B. dorsalis* were clustered together, with high sequence similarity to the *Hsp23* group from other species*.*

### 3.5 Transcriptome verification of *Hsp23* in* B. correcta* and* B. dorsalis*

**Figure 7:**
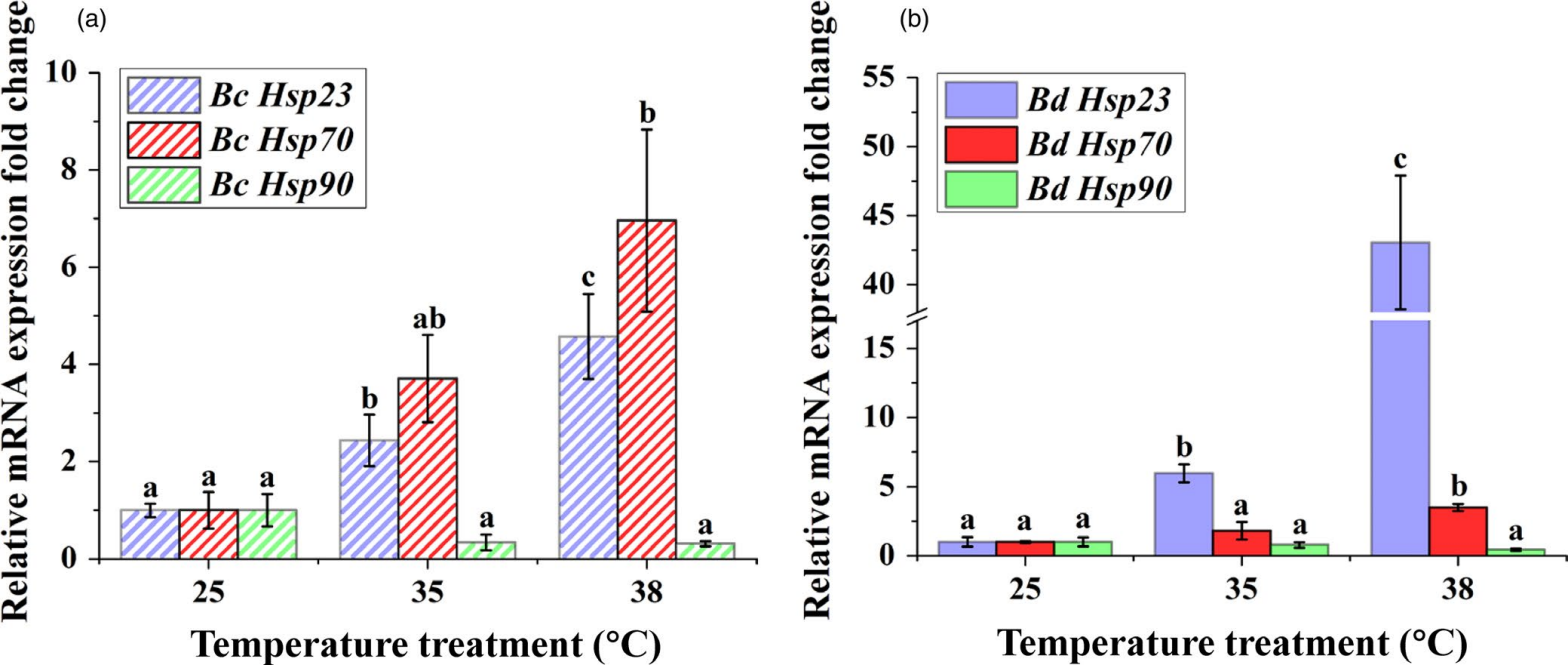
Expression of *Hsp23, Hsp70* and *Hsp90* at two hardening temperatures (35 and 38°C) and the 25°C control in *Bactrocera correcta* (Bc) and* B. dorsalis* (Bd). (a) Expression of *Hsp23*, *Hsp70* and *Hsp90* in *B. correcta*. (b) Expression of *Hsp23*, *Hsp70* and *Hsp90* in *B. dorsalis*. *18s* was used as the control gene. Different letters above the bars represent significant differences at *p* < 0.05, as determined by a *t* test

Compared with other two *Hsps*, *Hsp70* and *Hsp90,* that are potentially related to temperature adaptation in *B. correcta* and *B. dorsalis*, *Hsp23* increased significantly at the two hardening temperatures and had expression patterns across samples in these two species consistent with the transcriptome results (Figure 7; Hu et al., 2014). The expression level of *Hsp23* was similar under 35°C in two species but showed a difference at 38°C. Compared to the control group, the fold change in *B. correcta* was 2.4 and 4.6 times for 35 and 38°C, respectively (Figure 7a). This compared to fold changes in *B. dorsalis* of 5.3 and 43.1 times, respectively (Figure 7b). In these two species, the other two *Hsps* had the similar expression pattern; *Hsp70* expression was increased at 38°C but there was no change in *Hsp90* at the two hardening temperatures (Figure 7).

### 3.6 Gene function

**Figure 8:**
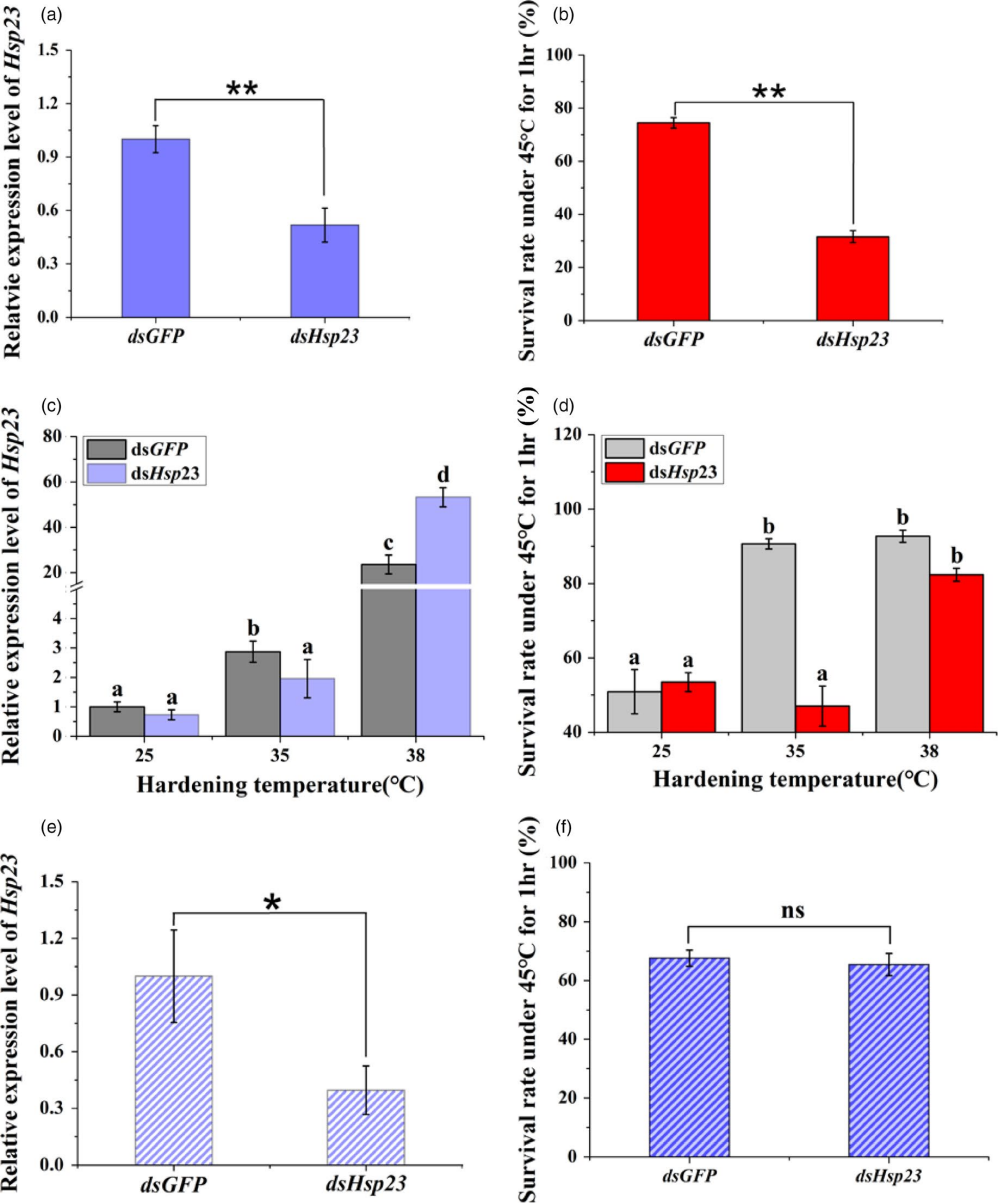
Functional analysis of *Hsp23* using RNAi in *Bactrocera dorsalis* and *B. correcta*. (a) Expression of *Hsp23* after RNAi in* B. dorsalis*. (b) Survival rate of *B. dorsalis* under the extreme thermal stress of 45°C for 1 hr. (c) Expression of *Hsp23* after heat hardening temperatures in* B. dorsalis*. (d) Survival rate of dsRNA‐feeding larvae exposed to extreme thermal stress 45°C after heat hardening treatments in* B. dorsalis*. (e) Expression of *Hsp23* after RNAi in *B. correcta*. (f) Survival rate of *B. correcta* under extreme thermal stress 45°C for 1 hr. All the flies of *B. correcta* and *B. dorsalis* were fed by ds*Hsp23* and ds*GFP* for 96 hr. *18s* was used as the control gene. The letter “*” and “**” above the bars represent significant differences at *p* < 0.05 and *p* < 0.01, respectively, and “ns” represents no significant differences. Different letters above indicate significant differences at *p* < 0.05, as determined by a t or Tukey HSD test

*Bactrocera dorsalis* has been suggested to have a stronger invasion ability compared with other tephritid fruit flies, and we chose *Hsp23* in this species to study whether this gene influenced temperature adaptation and caused the difference between these two species (Liu et al., 2017; Malacrida et al., 2007). Despite the similar expression pattern and sequence, the gene showed distinct functions between these two species. Therefore, an exposure of 3rd late‐instar larvae was used to study whether low expression of *Hsp23* could decrease survival. Compared with the ds*GFP*‐feeding group, ds*Hsp23* exposure led to a 0.48‐fold reduction in expression in *B. dorsalis* (Figure 8a) while survival rate under 45°C was decreased by 42.9% (Figure 8b). The result suggests that constitutive expression of *Hsp23* increases survival of *B. dorsalis* under heat. We used two hardening temperatures of 35 and 38°C to study whether suppressed *Hsp23* expression influenced survival rate following hardening. In the ds*Hsp23*‐feeding treatments, compared to the 25°C group, fold change expression of *Hsp23* in *B. dorsalis* increased by 1.96 and 83.27 times for the 35 and 38°C treatments, respectively (Figure 8c), while survival rate rose to 47.05% and 82.36%, respectively (Figure 8d). For the ds*GFP*‐feeding group, the fold change expression of *Hsp23* in *B. dorsalis* was 2.87 and 23.61 times for 35 and 38°C, respectively (Figure 8c), and the survival rate rose to 90.65% and 92.71% for 35 and 38°C, respectively (Figure 8d). The survival rate of the ds*GFP* group therefore increased significantly under both hardening temperatures compared to the control groups. But for the ds*Hsp23*‐feeding group, compared to the treatments under 25°C, survival rate only increased under 38°C when expression level of the target gene also significantly increased. For *B. correcta,* although the target gene *Hsp23* showed a 0.60‐fold decrease in expression (Figure 8e), survival rate under 45°C for 1 hr was not affected (Figure 8f).

## 4 DISCUSSION

### 4.1 Species differences in hardening

We showed that while both *Bactrocera* species were hardened by nonlethal temperatures, the temperatures range of 37–40°C led to a stronger hardening response for *B. dorsalis,* while *B. correcta* performed better following exposure to 34–36°C (Figure 2). The more narrowly distributed *B. correcta* seemed to show a heat hardening response more rapidly than the more widely distributed species. These results highlight differences among species in hardening responses which has also been noted in other groups of related species, particularly in *Drosophila* (Malmendal et al., 2006; Nyamukondiwa et al., 2011; Overgaard, Sørensen, Com, & Colinet, 2014; Willot et al., 2017). They also appear to be consistent with previous studies on temperature responses of other life stages of these two species (Hu et al., 2014; Liu & Ye, 2009; Pieterse et al., 2017). For *B. dorsalis,* heat shock tolerance of larvae was significantly enhanced by exposing to heat hardening 37 and 39°C for 1 or 2 hr (Hu et al., 2014; Pieterse et al., 2017). In *B. correcta,* the temperatures from 30 to 33°C appeared to be the most suitable for egg, larva and pupa development, and the preoviposition time was shortened following exposure to 33–36°C (Liu & Ye, 2009).

Whether these differences in hardening responses contribute to differences in the distribution of the two species is unclear. Other comparisons of insects also provide mixed evidence for associations between hardening and species distributions. In a comparison of the widely distributed *C. capitata* with its more narrowly distributed relative *C. rosa*, both species show similar levels of survival under acute high and low temperatures exposures when reared under common conditions and with a pretreatment of 36°C (1 hr) which altered survival at 41°C (Nyamukondiwa, Kleynhans, & Terblanche, 2010). Rapid cold hardening ability has been linked to species distributions in Collembola, and heat hardening has been related to different distributions in two *Cataglyphis* ants (Bahrndorff, Loeschcke, Pertoldi, Beier, & Holmstrup, 2009; Willot et al., 2017), but there is often no link between hardening and species distributions in *Drosophila* (Overgaard, Kristensen, Mitchell, & Hoffmann, 2011). Apart from hardening responses, other environmental factors can also influence the distribution and abundance of tephritid species (Vayssières, Carel, Coubes, & Duyck, 2008). Some factors affect the fitness of fruit flies and have an indirect influence on their distribution, while others have a direct influence. It was found that high temperatures, low humidity and the absence of suitable host fruits for oviposition could cause ovarian immaturity thus influence further spread and distribution of *Bactrocera* species (De Meyer et al., 2010). Due to the preference for certain hosts, *Dacus ciliatus* enhances its biotic potentialities and maintains its population at low levels especially at low altitudes to avoid competition with the melon fly *B. cucurbitae* (Vayssières et al., 2008).

For *Bactrocera* species, compared to *B. dorsalis*, *B. correcta* survival was more stable across all the high temperatures, which meant this species was not as sensitive as *B. dorsalis* to the hardening temperatures. The transcriptome results, including the number of regulated genes and pathways, also showed the stability of hardening response. The stable environment in the distribution range of *B. correcta* such as Yunnan, China, might contribute to this situation*.* Stability of transcription might bring some advantages such as a stable metabolic rate when temperature ranges around mild‐high levels, especially from 34 to 36°C. This stability may facilitate the ability of *B. correcta* to invade into areas that lack extreme heat. Given its rapid hardening response reflected by survival rate, *B. dorsalis* may be able to invade wider areas, especially under the climate change.

### 4.2 Gene expression patterns under two hardening temperatures

Elevated temperature exposure elicited a significant change in gene expression profiles between *B. dorsalis* and* B. correcta*. Many of the differences we identified correspond to specific genes or functional categories that have relevance to thermal tolerance in these two species, including genes such as *Hsps*, antioxidants/oxidative stress enzymes and pathways like lysosome, glycolysis, carbon metabolism and biosynthesis of amino acids as mentioned in the Results. In the PCA analysis and the comparison of up‐ and downregulated genes (Figure 3), it appears that the transcriptome of *B. dorsalis* is more responsive to high temperature than *B. correcta*, especially under 38°C, which contributed to a more variable survival rate. Interestingly, *B. correcta* shows a more rapid hardening response yet its transcriptome is not as responsive and the genes in *B. correcta* regulated more stably, which was consistent with survival rate. The different responses between species were also evident in our GO and KEGG analysis (Supporting Information Figures S3 and S4), with the regulated genes in *B. correcta* annotated with protein synthesis that could protect organisms against heat and oxidative stresses by eliminating hydrogen peroxide (Lu, Bai, Zheng, & Lu, 2017), whereas in *B. dorsalis,* changes to genes involved in a range of metabolic processes such as carbon metabolism were involved under hardening temperatures (Figure 4).

When considering both species, the response to stimulus was one of the most significantly enriched GO terms considering the DEG number and ratio of DEGs of the inquiry set in all related genes of the reference set (Supporting Information Figure S3). *Hsps* were the largest family to respond to stimulus among all the DEGs, with the highest levels of expression (Figure 5). Among all the genes upregulated in two hardening temperatures, the *Hsp*s were the only ones which were clearly candidates for the hardening process. Despite differences among species in some groups of genes, both species showed a number of upregulated *Hsp* genes associated with the hardening response under 38°C (Figures 5 and 6a,c). However, in *B. dorsalis,* most *Hsp* genes were downregulated at 35°C except *Hsp23.* We checked heat shock factors and thermal receptors including transient receptor potential (TRP), ion transporter, Gr28b.d and Na^+^/ K^+^‐ATPase in the list of differentially expressed genes. Only one Na^+^P‐type‐ATPase was found downregulated in the two *Bactrocera* species at 35°C, which may reflect a decrease of Na^+^ transportation and signaling transduction. Such differences may contribute to different hardening responses of the species at the two temperatures*.* In other studies using related species, differences in expression level of certain genes in response to the same environmental conditions have also been noted, including in fish, corals and planthoppers (Barshis et al., 2013; Huang et al., 2017; Wellband & Heath, 2017).

### 4.3 *Hsp23* expression and heat tolerance

We showed that suppressing *Hsp23* expression decreased the hardening response of *B. dorsalis*. However, RNAi induced different responses between *B. dorsalis* and *B. correcta*. In many studies, *Hsp70* has been suggested essential to heat tolerance (Bahrndorff, Mariën, Loeschcke, & Ellers, 2009; Bettencourt, Hogan, Nimali, & Drohan, 2008; Dahlgaard et al., 1998; Sørensen & Loeschcke, 2001; Zizzari & Ellers, 2011). However, in our study, we found that expression of *Hsp23* changed more rapidly and more intensely than *Hsp70* in* B. dorsalis,* which might be caused by s*Hsp*s at low *Hsp70* concentrations inhibiting the disaggregation and refolding processes (Żwirowski et al., 2017). As s*Hsp*s act as the first line of defense and are the key factors in modifying protein aggregation, the rapid expression of *Hsp23* indicates that the s*Hsp* can respond to temperature change more quickly than other *Hsps* (Żwirowski et al., 2017).

While *Hsp23* which was not the most expressed gene in *B. correcta*, it was chosen for functional studies because of the strong invasion ability in* B. dorsalis* where it was the most expressed gene, and considered in both species because of high similarities in expression pattern and sequences (Figure 7). The *sHsps* play important roles in many key physiological activities such as protein folding and transportation, embryo development, and immunization mechanisms (Li et al., 2009). In a recent study, binding by these proteins has been suggested being important mechanisms for protecting other cellular proteins from denaturation under thermal stress and other stresses, and overexpression of *sHsps* can enhance the tolerance of cells to temperature changes (Li et al., 2009; Wang et al., 2017). Among *sHsps*, *Hsp23* shows a key role in temperature adaptation in many species. In *Drosophila melanogaster,* gene knockdown experiments have suggested that *Hsp22* and *Hsp23* genes contribute to adaptive responses to fluctuating thermal conditions and particularly in chill coma recovery (Colinet, Lee, & Hoffmann, 2010). Also, overexpression of *Hsp23* muscle‐specific has been suggested to promote proteostasis and protect muscle from heat stress (Kawasaki et al., 2016). Moreover, the overexpression of muscle‐specific *Hsp23* gene in female ovaries produced offspring embryos with increased thermal tolerance (Lockwood, Julick, & Montooth, 2017). In other species such as *Leishmania donovani,* the scuttle fly *Megaselia scalaris* and the whitefly *Bemisia tabaci, Hsp23* expression is required for surviving extreme temperature treatments (Díaz et al., 2015; Hombach, Ommen, MacDonald, & Clos, 2014; Malewski et al., 2015).

We used hardening temperatures 35 and 38°C to increase the expression of *Hsp23.* Although we showed an effect of *Hsp23* expression knockdown on the heat tolerance and hardening of *B. dorsalis*, we did not find a similar effect in *B. correct* on high temperature tolerance. Perhaps a higher level of knockdown is required to trigger an effect of this gene on tolerance in *B. correcta*, particularly if the gene has multiple functions and there is a cost of gene expression associated with hardening on other fitness components (Chen, Feder, & Kang, 2018; Sørensen et al., 2003). It is also possible that this gene has no impact on the hardening response of *B. correcta*, despite the substantial upregulation of this gene under the hardening treatment. We did not consider the association between this gene and the hardening response in *B. correcta* for three reasons. Firstly, despite a high level of sequence conservation between the species, the thermal responses did not decrease at 45°C even with successful knockdown as we mentioned in the methods, suggesting that *Hsp23* has limited or no effect on heat tolerance (Figure 8). Secondly, according to the similar expression level of *Hsp23* induced at 35°C in *B. dorsalis* and* B. correcta,* two hardening temperatures (35 and 38°C) after the RNAi in *B. correcta* might not recover the expression of this gene (Figures 7 and 8)*.* Finally, although *Hsp23* showed an increased expression level, there was no significant difference in survival rate between 35 and 38°C (Figures 2 and 7a).

Ambient temperature is a key environmental factor influencing the ecology and evolution of ectotherms, and invasive species can be used as models for investigating issues related to adaptive processes under changing ambient conditions and bringing ecological and evolutionary processes into a common framework (Bennett & Lenski, 2007; Diamond, Chick, Perez, Strickler, & Martin, 2017; Ghalambor, McKay, Carroll, & Reznick, 2007). In our research, despite being similar, these two invasive species differ in many respects such as heat hardening profiles, transcriptome responses and *Hsp* expression. These differences likely mediate thermal hardening and involve genes such as *Hsps* and pathways such as energy metabolism and biosynthesis. As more genes and pathways are regulated in *B. dorsalis,* this species may be more adaptable under high temperature stress.

## 5 CONCLUSIONS

It is important to understand the role of genes such as *Hsp*s and pathways in relation to the hardening response and stress resistance, in order to develop a mechanistic basis to evolutionary change and plastic responses (Sørensen et al., 2003). In our study, we used two successful invasive species that have invaded different thermal environments to understand the role of hardening and plasticity in climate adaptation. Based on our results, the *Bactrocera* species have a varied ability to adapt to temperatures. Different hardening responses may be related to the regulation of certain genes like *Hsp23* which in turn could contribute to species distributions and invasive potential. The widely distributed species, *B. dorsalis*, seems more sensitive to temperature change at the transcriptome level. This sensitivity may help adaptive thermal responses, whereas *B. correcta* might be at an advantage in milder environments. However, this study only provides a starting point for understanding the genomic basis of climate adaptation in invasive fruit flies and the functional studies in particular need to be expanded to other gene families. Eventually, these types of studies may indicate targets for genetic manipulation to eventually control these pests. For instance, the importance of *Hsp23* in heat hardening in *B. dorsalis* may point to this gene as being a useful candidate for gene drive technology to suppress this pest at warm times of the year.

## CONFLICT OF INTEREST

None declared.

## Data Availability

RNA‐Seq data are available at National Center for Biotechnology Information (NCBI): Sequence Read Archives SRP158095 and BioProject accession PRJNA486250. The data sets supporting the conclusions of this article are included within the Supporting Information files.

## References

[eva12793-bib-0001] Alexa, A. , & Rahnenfuhrer, J. (2010). topGO: Enrichment analysis for gene ontology. R package version 2.22. Vienna, Austria: R Foundation for Statistical Computing Vienna.

[eva12793-bib-0002] Altschul, S. F. , Madden, T. L. , Schäffer, A. A. , Zhang, J. , Zhang, Z. , Miller, W. , & Lipman, D. J. (1997). Gapped BLAST and PSI‐BLAST: A new generation of protein database search programs. Nucleic Acids Research, 25(17), 3389–3402. 10.1093/nar/25.17.3389 9254694 PMC146917

[eva12793-bib-0003] Anders, S. , & Huber, W. (2010). Differential expression analysis for sequence count data. Genome Biology, 11(10), R106 10.1186/gb-2010-11-10-r106 20979621 PMC3218662

[eva12793-bib-0004] Ashburner, M. , Ball, C. A. , Blake, J. A. , Botstein, D. , Butler, H. , Cherry, J. M. , … Sherlock, G. (2000). Gene ontology: Tool for the unification of biology. Nature Genetics, 25(1), 25 10.1038/75556 10802651 PMC3037419

[eva12793-bib-0005] Bahrndorff, S. , Loeschcke, V. , Pertoldi, C. , Beier, C. , & Holmstrup, M. (2009). The rapid cold hardening response of *Collembola* is influenced by thermal variability of the habitat. Functional Ecology, 23(2), 340–347.

[eva12793-bib-0006] Bahrndorff, S. , Mariën, J. , Loeschcke, V. , & Ellers, J. (2009). Dynamics of heat‐induced thermal stress resistance and hsp70 expression in the springtail, *Orchesella cincta* . Functional Ecology, 23(2), 233–239.

[eva12793-bib-0007] Bairoch, A. , Apweiler, R. , Wu, C. H. , Barker, W. C. , Boeckmann, B. , Ferro, S. , … Martin, M. J. (2005). The universal protein resource (UniProt). Nucleic Acids Research, 33(suppl_1), D154–D159. 15608167 10.1093/nar/gki070 PMC540024

[eva12793-bib-0008] Barshis, D. J. , Ladner, J. T. , Oliver, T. A. , Seneca, F. O. , Traylor‐Knowles, N. , & Palumbi, S. R. (2013). Genomic basis for coral resilience to climate change. Proceedings of the National Academy of Sciences, 110(4), 1387–1392. 10.1073/pnas.1210224110 PMC3557039 23297204

[eva12793-bib-0009] Bennett, A. F. , & Lenski, R. E. (2007). An experimental test of evolutionary trade‐offs during temperature adaptation. Proceedings of the National Academy of Sciences, 104(suppl 1), 8649–8654. 10.1073/pnas.0702117104 PMC1876442 17494741

[eva12793-bib-0010] Bettencourt, B. R. , Hogan, C. C. , Nimali, M. , & Drohan, B. W. (2008). Inducible and constitutive heat shock gene expression responds to modification of *Hsp70* copy number in *Drosophila melanogaster* but does not compensate for loss of thermotolerance in *Hsp70* null flies. BMC Biology, 6(1), 5 10.1186/1741-7007-6-5 18211703 PMC2257928

[eva12793-bib-0011] Bolger, A. M. , Lohse, M. , & Usadel, B. (2014). Trimmomatic: A flexible trimmer for Illumina sequence data. Bioinformatics, 30(15), 2114–2120. 10.1093/bioinformatics/btu170 24695404 PMC4103590

[eva12793-bib-0012] Borchel, A. , Komisarczuk, A. Z. , Rebl, A. , Goldammer, T. , & Nilsen, F. (2018). Systematic identification and characterization of stress‐inducible heat shock proteins (HSPs) in the salmon louse (*Lepeophtheirus salmonis*). Cell Stress and Chaperones, 23(1), 127–139. 10.1007/s12192-017-0830-9 28695332 PMC5741587

[eva12793-bib-0013] Chen, B. , Feder, M. E. , & Kang, L. (2018). Evolution of heat‐shock protein expression underlying adaptive responses to environmental stress. Molecular Ecology, 27(15), 3040–3054. 10.1111/mec.14769 29920826

[eva12793-bib-0014] Chen, B. , & Wagner, A. (2012). *Hsp90* is important for fecundity, longevity, and buffering of cryptic deleterious variation in wild fly populations. BMC Evolutionary Biology, 12(1), 25 10.1186/1471-2148-12-25 22369091 PMC3305614

[eva12793-bib-0015] Clarke, A. (2003). Costs and consequences of evolutionary temperature adaptation. Trends in Ecology & Evolution, 18(11), 573–581. 10.1016/j.tree.2003.08.007

[eva12793-bib-0016] Colinet, H. , Lee, S. F. , & Hoffmann, A. (2010). Functional characterization of the Frost gene in *Drosophila melanogaster*: Importance for recovery from chill coma. PLoS ONE, 5(6), e10925 10.1371/journal.pone.0010925 20532197 PMC2880008

[eva12793-bib-0017] Dahlgaard, J. , Loeschcke, V. , Michalak, P. , & Justesen, J. (1998). Induced thermotolerance and associated expression of the heat‐shock protein *Hsp70* in adult *Drosophila melanogaster* . Functional Ecology, 12(5), 786–793. 10.1046/j.1365-2435.1998.00246.x

[eva12793-bib-0018] David, J. R. , Gibert, P. , Gravot, E. , Petavy, G. , Morin, J. P. , Karan, D. , & Moreteau, B. (1997). Phenotypic plasticity and developmental temperature in *Drosophila:* Analysis and significance of reaction norms of morphometrical traits. Journal of Thermal Biology, 22(6), 441–451. 10.1016/S0306-4565(97)00063-6

[eva12793-bib-0019] De Meyer, M. , Robertson, M. P. , Mansell, M. W. , Ekesi, S. , Tsuruta, K. , Mwaiko, W. , … Peterson, A. T. (2010). Ecological niche and potential geographic distribution of the invasive fruit fly *Bactrocera invadens* (Diptera, Tephritidae). Bulletin of Entomological Research, 100(1), 35–48. 10.1017/S0007485309006713 19323851

[eva12793-bib-0020] Delpuech, J. M. , Moreteau, B. , Chiche, J. , Pla, E. , Vouidibio, J. , & David, J. R. (1995). Phenotypic plasticity and reaction norms in temperate and tropical populations of *Drosophila melanogaster*: Ovarian size and developmental temperature. Evolution, 49(4), 670–675. 28565134 10.1111/j.1558-5646.1995.tb02303.x

[eva12793-bib-0021] Deng, Y. , Li, J. , Wu, S. , Zhu, Y. , Chen, Y. , & He, F. (2006). Integrated nr database in protein annotation system and its localization. Computer Engineering, 32(5), 71–74.

[eva12793-bib-0022] Diamond, S. E. , Chick, L. , Perez, A. , Strickler, S. A. , & Martin, R. A. (2017). Rapid evolution of ant thermal tolerance across an urban‐rural temperature cline. Biological Journal of the Linnean Society, 121(2), 248–257. 10.1093/biolinnean/blw047

[eva12793-bib-0023] Díaz, F. , Orobio, R. F. , Chavarriaga, P. , & Toro‐Perea, N. (2015). Differential expression patterns among heat‐shock protein genes and thermal responses in the whitefly *Bemisia tabaci* (MEAM 1). Journal of Thermal Biology, 52, 199–207. 10.1016/j.jtherbio.2015.07.004 26267515

[eva12793-bib-0024] DiDomenico, B. J. , Bugaisky, G. E. , & Lindquist, S. (1982). Heat shock and recovery are mediated by different translational mechanisms. Proceedings of the National Academy of Sciences, 79(20), 6181–6185. 10.1073/pnas.79.20.6181 PMC347083 6815647

[eva12793-bib-0025] Dou, W. , Tian, Y. , Liu, H. , Shi, Y. , Smagghe, G. , & Wang, J. J. (2017). Characteristics of six small heat shock protein genes from *Bactrocera dorsalis*: Diverse expression under conditions of thermal stress and normal growth. Comparative Biochemistry and Physiology Part B: Biochemistry and Molecular Biology, 213, 8–16. 10.1016/j.cbpb.2017.07.005 28735974

[eva12793-bib-0026] Ferreira, J. , & Zwinderman, A. (2006). On the Benjamini‐Hochberg method. Annals of Statistics, 34(4), 1827–1849. 10.1214/009053606000000425

[eva12793-bib-0027] Finn, R. D. , Bateman, A. , Clements, J. , Coggill, P. , Eberhardt, R. Y. , Eddy, S. R. , … Mistry, J. (2013). Pfam: The protein families database. Nucleic Acids Research, 42(D1), D222–D230. 24288371 10.1093/nar/gkt1223 PMC3965110

[eva12793-bib-0028] Fu, L. , Niu, B. , Zhu, Z. , Wu, S. , & Li, W. (2012). CD‐HIT: Accelerated for clustering the next‐generation sequencing data. Bioinformatics, 28(23), 3150–3152. 10.1093/bioinformatics/bts565 23060610 PMC3516142

[eva12793-bib-0029] Ghalambor, C. K. , McKay, J. K. , Carroll, S. P. , & Reznick, D. N. (2007). Adaptive versus non‐adaptive phenotypic plasticity and the potential for contemporary adaptation in new environments. Functional Ecology, 21(3), 394–407. 10.1111/j.1365-2435.2007.01283.x

[eva12793-bib-0030] Gibert, P. , Hill, M. , Pascual, M. , Plantamp, C. , Terblanche, J. S. , Yassin, A. , & Sgrò, C. M. (2016). *Drosophila* as models to understand the adaptive process during invasion. Biological Invasions, 18(4), 1089–1103. 10.1007/s10530-016-1087-4

[eva12793-bib-0031] Grabherr, M. G. , Haas, B. J. , Yassour, M. , Levin, J. Z. , Thompson, D. A. , Amit, I. , … Regev, A. (2011). Full‐length transcriptome assembly from RNA‐Seq data without a reference genome. Nature Biotechnology, 29(7), 644–652. 10.1038/nbt.1883 PMC3571712 21572440

[eva12793-bib-0032] Guo, S. , Zhao, Z. , Liu, L. , Li, Z. , & Shen, J. (2018). Comparative transcriptome analyses uncover key candidate genes mediating flight capacity in *Bactrocera dorsalis* (Hendel) and *Bactrocera correcta* (Bezzi) (Diptera: Tephritidae). International Journal of Molecular Sciences, 19(2), 396. 10.3390/ijms19020396 PMC5855618 29385681

[eva12793-bib-0033] Haas, B. J. , Papanicolaou, A. , Yassour, M. , Grabherr, M. , Blood, P. D. , Bowden, J. , … Regev, A. (2013). *De novo* transcript sequence reconstruction from RNA‐seq using the Trinity platform for reference generation and analysis. Nature Protocols, 8(8), 1494 10.1038/nprot.2013.084 23845962 PMC3875132

[eva12793-bib-0034] Hallman, G. J. , Myers, S. W. , El‐Wakkad, M. F. , Tadrous, M. D. , & Jessup, A. J. (2013). Development of phytosanitary cold treatments for oranges infested with *Bactrocera invadens* and *Bactrocera zonata* (Diptera: Tephritidae) by comparison with existing cold treatment schedules for *Ceratitis capitata* (Diptera: Tephritidae). Journal of Economic Entomology, 106(4), 1608–1612. 24020272 10.1603/ec13066

[eva12793-bib-0035] Hoffmann, A. A. , & Ross, P. A. (2018). Rates and patterns of laboratory adaptation in (mostly) insects. Journal of Economic Entomology, 111(2), 501–509. 10.1093/jee/toy024 29506036

[eva12793-bib-0036] Hoffmann, A. A. , Sørensen, J. G. , & Loeschcke, V. (2003). Adaptation of *Drosophila* to temperature extremes: Bringing together quantitative and molecular approaches. Journal of Thermal Biology, 28(3), 175–216. 10.1016/S0306-4565(02)00057-8

[eva12793-bib-0037] Hombach, A. , Ommen, G. , MacDonald, A. , & Clos, J. (2014). A small heat shock protein is essential for thermotolerance and intracellular survival of *Leishmania donovani* . Journal of Cell Science, 127, 4762–4773. 10.1242/jcs.157297 25179594 PMC4215717

[eva12793-bib-0038] Hu, J. T. , Chen, B. , & Li, Z. H. (2014). Thermal plasticity is related to the hardening response of heat shock protein expression in two *Bactrocera* fruit flies. Journal of Insect Physiology, 67, 105–113. 10.1016/j.jinsphys.2014.06.009 24992713

[eva12793-bib-0039] Huang, H. J. , Xue, J. , Zhuo, J. C. , Cheng, R. L. , Xu, H. J. , & Zhang, C. X. (2017). Comparative analysis of the transcriptional responses to low and high temperatures in three rice planthopper species. Molecular Ecology, 26(10), 2726–2737. 10.1111/mec.14067 28214356

[eva12793-bib-0040] Huang, L. H. , & Kang, L. (2007). Cloning and interspecific altered expression of heat shock protein genes in two leafminer species in response to thermal stress. Insect Molecular Biology, 16(4), 491–500. 10.1111/j.1365-2583.2007.00744.x 17651238

[eva12793-bib-0041] Huerta‐Cepas, J. , Szklarczyk, D. , Forslund, K. , Cook, H. , Heller, D. , Walter, M. C. , … Kuhn, M. (2015). eggNOG 4.5: A hierarchical orthology framework with improved functional annotations for eukaryotic, prokaryotic and viral sequences. Nucleic Acids Research, 44(D1), D286–D293. 26582926 10.1093/nar/gkv1248 PMC4702882

[eva12793-bib-0042] Jang, E. B. (1991). Thermal death kinetics and heat tolerance in early and late third instars of the oriental fruit fly (Diptera: Tephritidae). Journal of Economic Entomology, 84(4), 1298–1303. 10.1093/jee/84.4.1298

[eva12793-bib-0043] Kanehisa, M. , Goto, S. , Kawashima, S. , Okuno, Y. , & Hattori, M. (2004). The KEGG resource for deciphering the genome. Nucleic Acids Research, 32(suppl_1), D277–D280. 10.1093/nar/gkh063 14681412 PMC308797

[eva12793-bib-0044] Kawasaki, F. , Koonce, N. L. , Guo, L. , Fatima, S. , Qiu, C. , Moon, M. T. , … Ordway, R. W. (2016). Small heat shock proteins mediate cell‐autonomous and‐nonautonomous protection in a *Drosophila* model for environmental‐stress‐induced degeneration. Disease Models & Mechanisms, 9(9), 953–964. 27483356 10.1242/dmm.026385 PMC5047692

[eva12793-bib-0045] King, A. M. , & MacRae, T. H. (2015). Insect heat shock proteins during stress and diapause. Annual Review of Entomology, 60, 59–75. 10.1146/annurev-ento-011613-162107 25341107

[eva12793-bib-0046] Klepsatel, P. , Gáliková, M. , Maio, N. , Huber, C. D. , Schlötterer, C. , & Flatt, T. (2013). Variation in thermal performance and reaction norms among populations of *Drosophila melanogaster* . Evolution, 67(12), 3573–3587. 24299409 10.1111/evo.12221

[eva12793-bib-0047] Langmead, B. , Trapnell, C. , Pop, M. , & Salzberg, S. L. (2009). Ultrafast and memory‐efficient alignment of short DNA sequences to the human genome. Genome Biology, 10(3), R25 10.1186/gb-2009-10-3-r25 19261174 PMC2690996

[eva12793-bib-0048] Li, B. , & Dewey, C. N. (2011). RSEM: Accurate transcript quantification from RNA‐Seq data with or without a reference genome. BMC Bioinformatics, 12(1), 323 10.1186/1471-2105-12-323 21816040 PMC3163565

[eva12793-bib-0049] Li, R. , Li, Y. , Kristiansen, K. , & Wang, J. (2008). SOAP: Short oligonucleotide alignment program. Bioinformatics, 24(5), 713–714. 10.1093/bioinformatics/btn025 18227114

[eva12793-bib-0050] Li, Y. , Wu, Y. , Chen, H. , Wu, J. , & Li, Z. (2012). Population structure and colonization of *Bactrocera dorsalis* (Diptera: Tephritidae) in China, inferred from mtDNA *COI* sequences. Journal of Applied Entomology, 136(4), 241–251. 10.1111/j.1439-0418.2011.01636.x

[eva12793-bib-0051] Li, Z. W. , Li, X. , Yu, Q. Y. , Xiang, Z. H. , Kishino, H. , & Zhang, Z. (2009). The small heat shock protein (*sHSP*) genes in the silkworm, *Bombyx mori*, and comparative analysis with other insect *sHSP* genes. BMC Evolutionary Biology, 9(1), 215 10.1186/1471-2148-9-215 19715580 PMC2745388

[eva12793-bib-0052] Liu, H. , Hou, B. , Zhang, C. , He, R. , Liang, F. , Gu, M. , … Ma, J. (2014). Oviposition preference and offspring performance of the oriental fruit fly *Bactrocera dorsalis* and guava fruit fly *B. correcta* (Diptera: Tephritidae) on six host fruits. Acta Ecologica Sinica, 9, 2274–2281.

[eva12793-bib-0053] Liu, H. , Zhang, C. , Hou, B. H. , Ou‐Yang, G. C. , & Ma, J. (2017). Interspecific competition between *Ceratitis capitata* and two *Bactrocera* spp. (Diptera: Tephritidae) evaluated via adult behavioral interference under laboratory conditions. Journal of Economic Entomology, 110(3), 1145–1155. 28334323 10.1093/jee/tox083

[eva12793-bib-0054] Liu, X. , Jin, Y. , & Ye, H. (2013). Recent spread and climatic ecological niche of the invasive guava fruit fly, *Bactrocera correcta,* in mainland China. Journal of Pest Science, 86(3), 449–458. 10.1007/s10340-013-0488-8

[eva12793-bib-0055] Liu, X. , & Ye, H. (2009). Effect of temperature on development and survival of *Bactrocera correcta* (Diptera: Tephritidae). Scientific Research and Essay, 4(5), 467–472.

[eva12793-bib-0056] Lockwood, B. L. , Julick, C. R. , & Montooth, K. L. (2017). Maternal loading of a small heat shock protein increases embryo thermal tolerance in *Drosophila melanogaster* . Journal of Experimental Biology, 220(23), 4492–4501. 29097593 10.1242/jeb.164848 PMC5769566

[eva12793-bib-0057] Lu, Y. , Bai, Q. , Zheng, X. , & Lu, Z. (2017). Expression and enzyme activity of catalase in *Chilo suppressalis* (Lepidoptera: Crambidae) is responsive to environmental stresses. Journal of Economic Entomology, 110(4), 1803–1812. 10.1093/jee/tox117 28419293

[eva12793-bib-0058] Lux, S. A. , Copeland, R. S. , White, I. M. , Manrakhan, A. , & Billah, M. K. (2003). A new invasive fruit fly species from the *Bactrocera dorsalis* (Hendel) group detected in East Africa. International Journal of Tropical Insect Science, 23(4), 355–361. 10.1017/S174275840001242X

[eva12793-bib-0059] Malacrida, A. , Gomulski, L. , Bonizzoni, M. , Bertin, S. , Gasperi, G. , & Guglielmino, C. (2007). Globalization and fruitfly invasion and expansion: The medfly paradigm. Genetica, 131(1), 1147 10.1007/s10709-006-9117-2 17111234

[eva12793-bib-0060] Malewski, T. , Bogdanowicz, W. , Durska, E. , Łoś, M. , Kamiński, M. , & Kowalewska, K. (2015). Expression profiling of heat shock genes in a scuttle fly *Megaselia scalaris* (Diptera, Phoridae). Journal of Experimental Zoology Part A: Ecological Genetics and Physiology, 323(10), 704–713. 10.1002/jez.1963 26477614

[eva12793-bib-0061] Malmendal, A. , Overgaard, J. , Bundy, J. G. , Sørensen, J. G. , Nielsen, N. C. , Loeschcke, V. , & Holmstrup, M. (2006). Metabolomic profiling of heat stress: Hardening and recovery of homeostasis in *Drosophila* . American Journal of Physiology‐Regulatory, Integrative and Comparative Physiology, 291(1), R205–R212. 10.1152/ajpregu.00867.2005 16469831

[eva12793-bib-0062] Manjunatha, H. , Rajesh, R. , & Aparna, H. (2010). Silkworm thermal biology: A review of heat shock response, heat shock proteins and heat acclimation in the domesticated silkworm, *Bombyx mori* . Journal of Insect Science, 10(1), 204. 21265618 10.1673/031.010.20401 PMC3029153

[eva12793-bib-0063] Matsumura, T. , Matsumoto, H. , & Hayakawa, Y. (2017). Heat stress hardening of oriental armyworms is induced by a transient elevation of reactive oxygen species during sublethal stress. Archives of Insect Biochemistry and Physiology, 96(3). 10.1002/arch.21421 28872705

[eva12793-bib-0064] Myers, S. W. , Cancio‐Martinez, E. , Hallman, G. J. , Fontenot, E. A. , & Vreysen, M. J. (2016). Relative tolerance of six *Bactrocera* (Diptera: Tephritidae) species to phytosanitary cold treatment. Journal of Economic Entomology, 109(6), 2341–2347. 27660425 10.1093/jee/tow206

[eva12793-bib-0065] Nyamukondiwa, C. , Kleynhans, E. , & Terblanche, J. S. (2010). Phenotypic plasticity of thermal tolerance contributes to the invasion potential of Mediterranean fruit flies (*Ceratitis capitata*). Ecological Entomology, 35(5), 565–575. 10.1111/j.1365-2311.2010.01215.x

[eva12793-bib-0066] Nyamukondiwa, C. , Terblanche, J. S. , Marshall, K. E. , & Sinclair, B. J. (2011). Basal cold but not heat tolerance constrains plasticity among *Drosophila* species (Diptera: Drosophilidae). Journal of Evolutionary Biology, 24(9), 1927–1938. 10.1111/j.1420-9101.2011.02324.x 21658189

[eva12793-bib-0067] Overgaard, J. , Kristensen, T. N. , Mitchell, K. A. , & Hoffmann, A. A. (2011). Thermal tolerance in widespread and tropical *Drosophila* species: Does phenotypic plasticity increase with latitude? American Naturalist, 178(S1), S80–S96. 10.1086/661780 21956094

[eva12793-bib-0068] Overgaard, J. , Sørensen, J. G. , Com, E. , & Colinet, H. (2014). The rapid cold hardening response of *Drosophila melanogaster:* Complex regulation across different levels of biological organization. Journal of Insect Physiology, 62, 46–53. 10.1016/j.jinsphys.2014.01.009 24508557

[eva12793-bib-0069] Papadopoulos, N. T. (2008). Mediterranean fruit fly, Ceratitis capitata (Wiedemann) (Diptera: Tephritidae) In Capinera J. L. (Ed.), *Encyclopedia of Entomology* (pp. 2318–2322). Dordrecht, The Netherlands: Springer.

[eva12793-bib-0070] Permpoon, R. , Aketarawong, N. , & Thanaphum, S. (2011). Isolation and characterization of Doublesex homologues in the *Bactrocera* species: *B. dorsalis* (Hendel) and *B. correcta* (Bezzi) and their putative promoter regulatory regions. Genetica, 139(1), 113–127. 20976560 10.1007/s10709-010-9508-2

[eva12793-bib-0071] Pieterse, W. , Terblanche, J. S. , & Addison, P. (2017). Do thermal tolerances and rapid thermal responses contribute to the invasion potential of *Bactrocera dorsalis* (Diptera: Tephritidae)? Journal of Insect Physiology, 98, 1147–6. 10.1016/j.jinsphys.2016.11.004 27845146

[eva12793-bib-0072] Qin, Y. , Ni, W. , Wu, J. , Zhao, Z. , Chen, H. , & Li, Z. (2015). The potential geographic distribution of *Bactrocera correcta* (Diptera: Tephrididae) in China based on eclosion rate model. Applied Entomology and Zoology, 50(3), 371–381.

[eva12793-bib-0073] Qin, Y. , Paini, D. R. , Wang, C. , Fang, Y. , & Li, Z. (2015). Global establishment risk of economically important fruit fly species (Tephritidae). PLoS ONE, 10(1), e0116424. 25588025 10.1371/journal.pone.0116424 PMC4294639

[eva12793-bib-0074] Reitz, S. R. , & Trumble, J. T. (2002). Competitive displacement among insects and arachnids. Annual Review of Entomology, 47(1), 435–465. 10.1146/annurev.ento.47.091201.145227 11729081

[eva12793-bib-0075] Ron, D. , & Walter, P. (2007). Signal integration in the endoplasmic reticulum unfolded protein response. Nature Reviews Molecular Cell Biology, 8(7), 519 10.1038/nrm2199 17565364

[eva12793-bib-0076] Schulze, S. K. , Kanwar, R. , Gölzenleuchter, M. , Therneau, T. M. , & Beutler, A. S. (2012). SERE: Single‐parameter quality control and sample comparison for RNA‐Seq. BMC Genomics, 13(1), 524 10.1186/1471-2164-13-524 23033915 PMC3534338

[eva12793-bib-0077] Shen, G. M. , Huang, Y. , Jiang, X. Z. , Dou, W. , & Wang, J. J. (2013). Effect of β‐cypermetherin exposure on the stability of nine housekeeping genes in *Bactrocera dorsalis* (Diptera: Tephritidae). Florida Entomologist, 442–450.

[eva12793-bib-0078] Sisodia, S. , & Singh, B. N. (2006). Effect of exposure to short‐term heat stress on survival and fecundity in *Drosophila ananassae* . Canadian Journal of Zoology, 84(6), 895–899.

[eva12793-bib-0079] Sørensen, J. G. , Kristensen, T. N. , & Loeschcke, V. (2003). The evolutionary and ecological role of heat shock proteins. Ecology Letters, 6(11), 1025–1037. 10.1046/j.1461-0248.2003.00528.x

[eva12793-bib-0080] Sørensen, J. , & Loeschcke, V. (2001). Larval crowding in *Drosophila melanogaster* induces *Hsp70* expression, and leads to increased adult longevity and adult thermal stress resistance. Journal of Insect Physiology, 47(11), 1301–1307. 10.1016/S0022-1910(01)00119-6 12770182

[eva12793-bib-0081] Sørensen, J. G. , Nielsen, M. M. , Kruhøffer, M. , Justesen, J. , & Loeschcke, V. (2005). Full genome gene expression analysis of the heat stress response in *Drosophila melanogaster* . Cell Stress & Chaperones, 10(4), 312–328. 10.1379/CSC-128R1.1 16333985 PMC1283958

[eva12793-bib-0082] Tamura, K. , Peterson, D. , Peterson, N. , Stecher, G. , Nei, M. , & Kumar, S. (2011). MEGA5: Molecular evolutionary genetics analysis using maximum likelihood, evolutionary distance, and maximum parsimony methods. Molecular Biology and Evolution, 28(10), 2731–2739. 10.1093/molbev/msr121 21546353 PMC3203626

[eva12793-bib-0083] Tatusov, R. L. , Galperin, M. Y. , Natale, D. A. , & Koonin, E. V. (2000). The COG database: A tool for genome‐scale analysis of protein functions and evolution. Nucleic Acids Research, 28(1), 33–36. 10.1093/nar/28.1.33 10592175 PMC102395

[eva12793-bib-0084] Vayssières, J. F. , Carel, Y. , Coubes, M. , & Duyck, P. F. (2008). Development of immature stages and comparative demography of two cucurbit‐attacking fruit flies in Reunion Island: *Bactrocera cucurbitae* and *Dacus ciliatus* (Diptera Tephritidae). Environmental Entomology, 37(2), 307–314. 18419901 10.1603/0046-225x(2008)37[307:doisac]2.0.co;2

[eva12793-bib-0085] Vázquez, D. P. , Gianoli, E. , Morris, W. F. , & Bozinovic, F. (2017). Ecological and evolutionary impacts of changing climatic variability. Biological Reviews, 92(1), 22–42. 10.1111/brv.12216 26290132

[eva12793-bib-0086] Wang, H.‐J. , Shi, Z.‐K. , Shen, Q.‐D. , Xu, C.‐D. , Wang, B. , Meng, Z.‐J. , … Wang, S. U. (2017). Molecular cloning and induced expression of six small heat shock proteins mediating cold‐hardiness in *Harmonia axyridis* (Coleoptera: Coccinellidae). Frontiers in Physiology, 8, 60 10.3389/fphys.2017.00060 28232804 PMC5299025

[eva12793-bib-0087] Wang, L. , Feng, Z. , Wang, X. , Wang, X. , & Zhang, X. (2009). DEGseq: An R package for identifying differentially expressed genes from RNA‐seq data. Bioinformatics, 26(1), 136–138. 10.1093/bioinformatics/btp612 19855105

[eva12793-bib-0088] Weldon, C. W. , Nyamukondiwa, C. , Karsten, M. , Chown, S. L. , & Terblanche, J. S. (2018). Geographic variation and plasticity in climate stress resistance among southern African populations of *Ceratitis capitata* (Wiedemann) (Diptera: Tephritidae). Scientific Reports, 8(1), 9849 10.1038/s41598-018-28259-3 29959431 PMC6026165

[eva12793-bib-0089] Wellband, K. W. , & Heath, D. D. (2017). Plasticity in gene transcription explains the differential performance of two invasive fish species. Evolutionary Applications, 10(6), 563–576. 10.1111/eva.12463 28616064 PMC5469171

[eva12793-bib-0090] Willot, Q. , Gueydan, C. , & Aron, S. (2017). Proteome stability, heat hardening and heat‐shock protein expression profiles in *Cataglyphis* desert ants. Journal of Experimental Biology, 220(9), 1721–1728. 28232398 10.1242/jeb.154161

[eva12793-bib-0091] Wos, G. , & Willi, Y. (2018). Thermal acclimation in *Arabidopsis lyrata*: Genotypic costs and transcriptional changes. Journal of Evolutionary Biology, 31(1), 123–135. 29134788 10.1111/jeb.13208

[eva12793-bib-0092] Xie, C. , Mao, X. , Huang, J. , Ding, Y. , Wu, J. , Dong, S. , … Wei, L. (2011). KOBAS 2.0: A web server for annotation and identification of enriched pathways and diseases. Nucleic Acids Research, 39(Suppl 2), W316–W322. 21715386 10.1093/nar/gkr483 PMC3125809

[eva12793-bib-0093] Yang, Y. , & Smith, S. A. (2013). Optimizing *de novo* assembly of short‐read RNA‐seq data for phylogenomics. BMC Genomics, 14(1), 328 10.1186/1471-2164-14-328 23672450 PMC3663818

[eva12793-bib-0094] Yuan, S. , Kong, Q. , Xiao, C. , Yang, S. , Sun, W. , Zhang, J. , & Li, Z. (2006). Introduction to two kinds of artificial diets for mass rearing of adult *Bactrocera dorsalis* (Hendel). Journal of Huazhong Agricultural University, 25, 371–374.

[eva12793-bib-0096] Zizzari, Z. V. , & Ellers, J. (2011). Effects of exposure to short‐term heat stress on male reproductive fitness in a soil arthropod. Journal of Insect Physiology, 57(3), 421–426. 10.1016/j.jinsphys.2011.01.002 21215753

[eva12793-bib-0097] Żwirowski, S. , Kłosowska, A. , Obuchowski, I. , Nillegoda, N. B. , Piróg, A. , Ziętkiewicz, S. , … Liberek, K. (2017). *Hsp70* displaces small heat shock proteins from aggregates to initiate protein refolding. EMBO Journal, 36(6), 783–796. 10.15252/embj.201593378 28219929 PMC5350560

