# Supplementary material for: A transcriptional and functional analysis of heat hardening in two invasive fruit fly species, Bactrocera dorsalis and Bactrocera correcta
Source: Evol Appl. 2019 Apr 10;12(6):1147–63. doi: 10.1111/eva.12793 (PMC6597872; doi:10.1111/eva.12793)
Supplement: Supplementary file 1 [file EVA-12-1147-s001.docx]

**
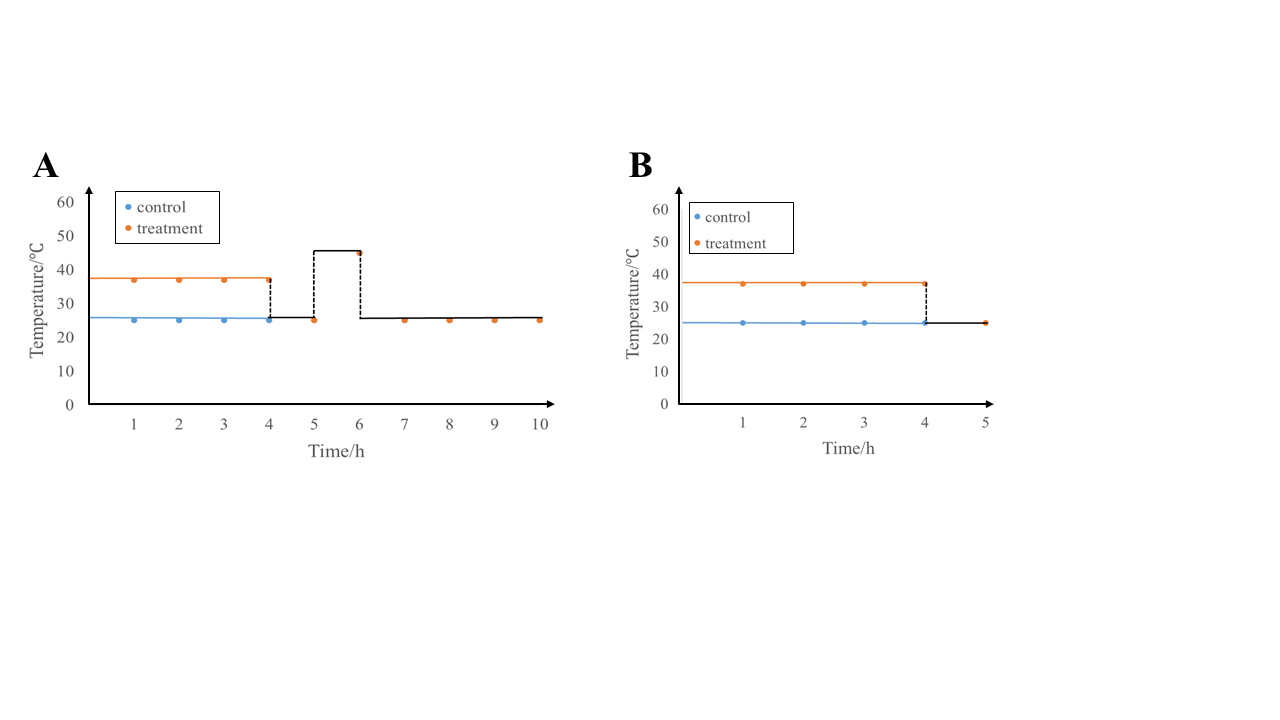
**

**S1. Experimental design.** A. Heat hardening treatments in *B. correcta* and *B. dorsalis*. B. Temperature treatments before transcriptome sequencing in *B. correcta* and *B. dorsalis*. The orange dots and lines represent a series of heat hardening temperatures (34-40°C) in heat-hardening groups. The blue dots and lines represent temperature treatments in the control group. The black dots and lines represent the same treatments applied to the heat-hardening and control groups.


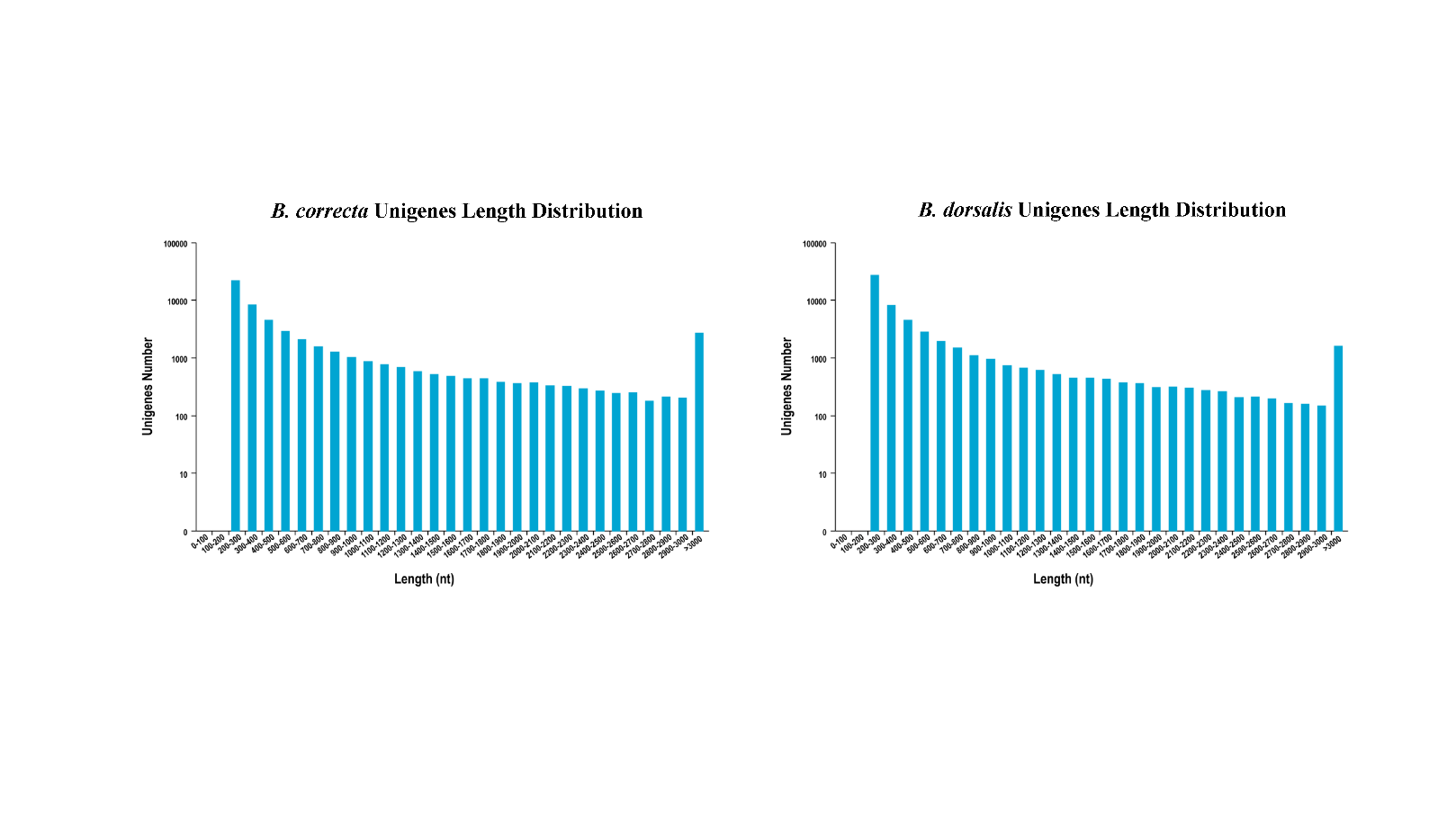


**S2. Unigenes length distribution of *B. correcta* and *B. dorsalis***.


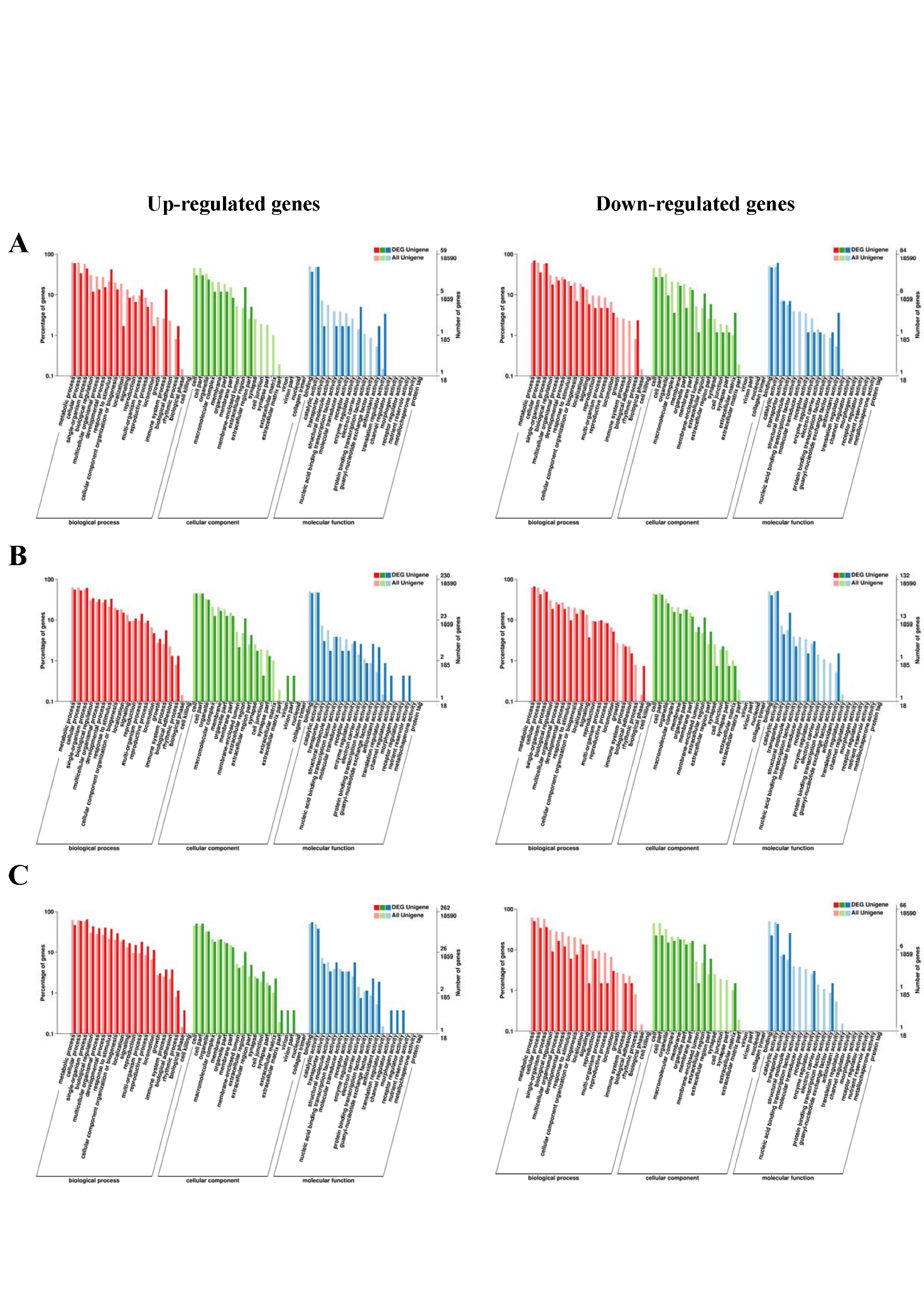


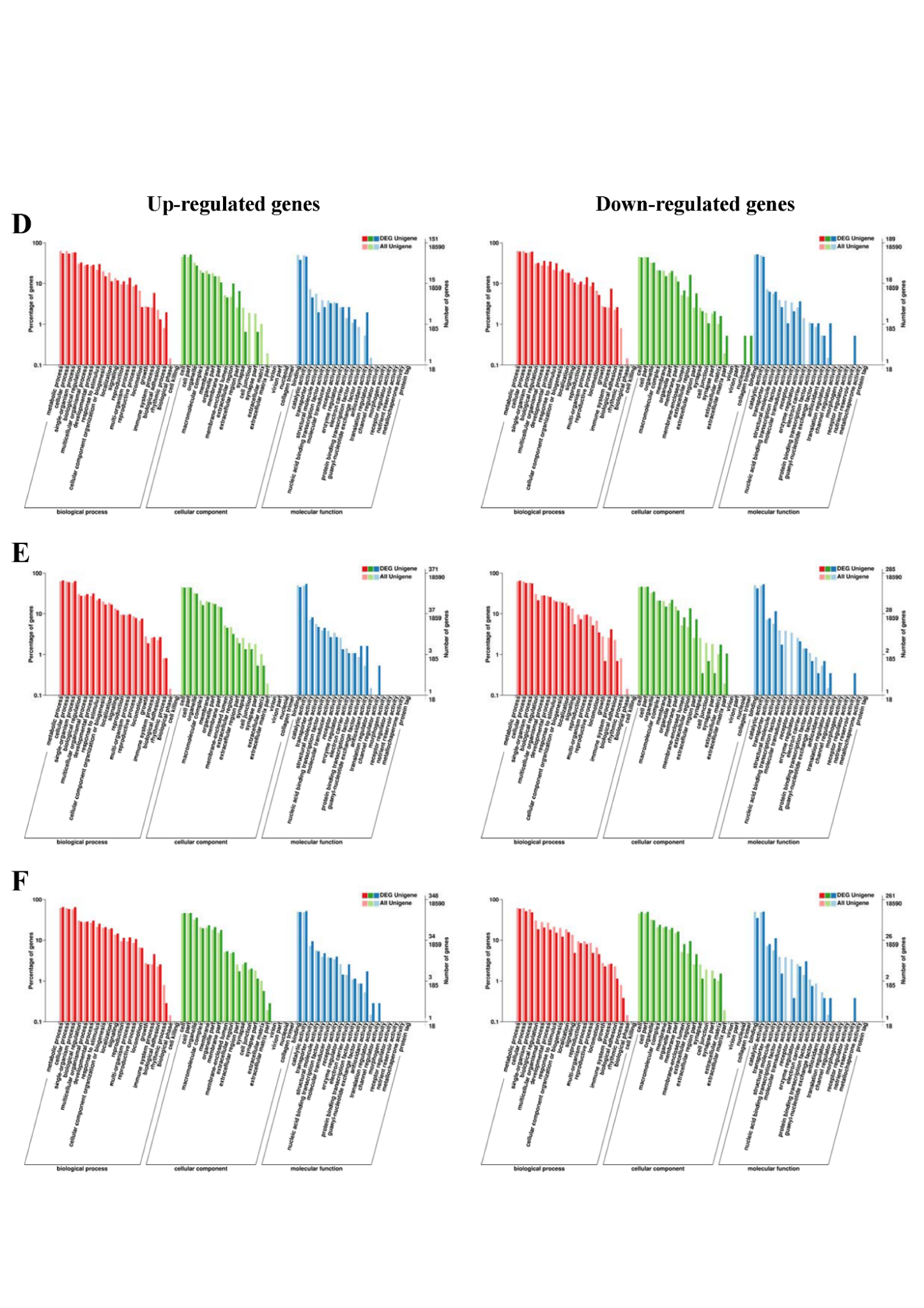
**S3. GO analysis of differentially expressed genes (DEGs) including up- and down-regulated genes with a fold change more than 2 in *B. correcta* and *B. dorsalis*.** A. 35 vs 25°C for *B. correcta.* B. 38 vs 25°C for *B. correcta.* C. 38 vs 35°C for *B. correcta.* D. 35 vs 25°C for *B. dorsalis.* E. 38 vs 25°C for *B. dorsalis.* F. 38 vs 35°C for *B. dorsalis.*

*
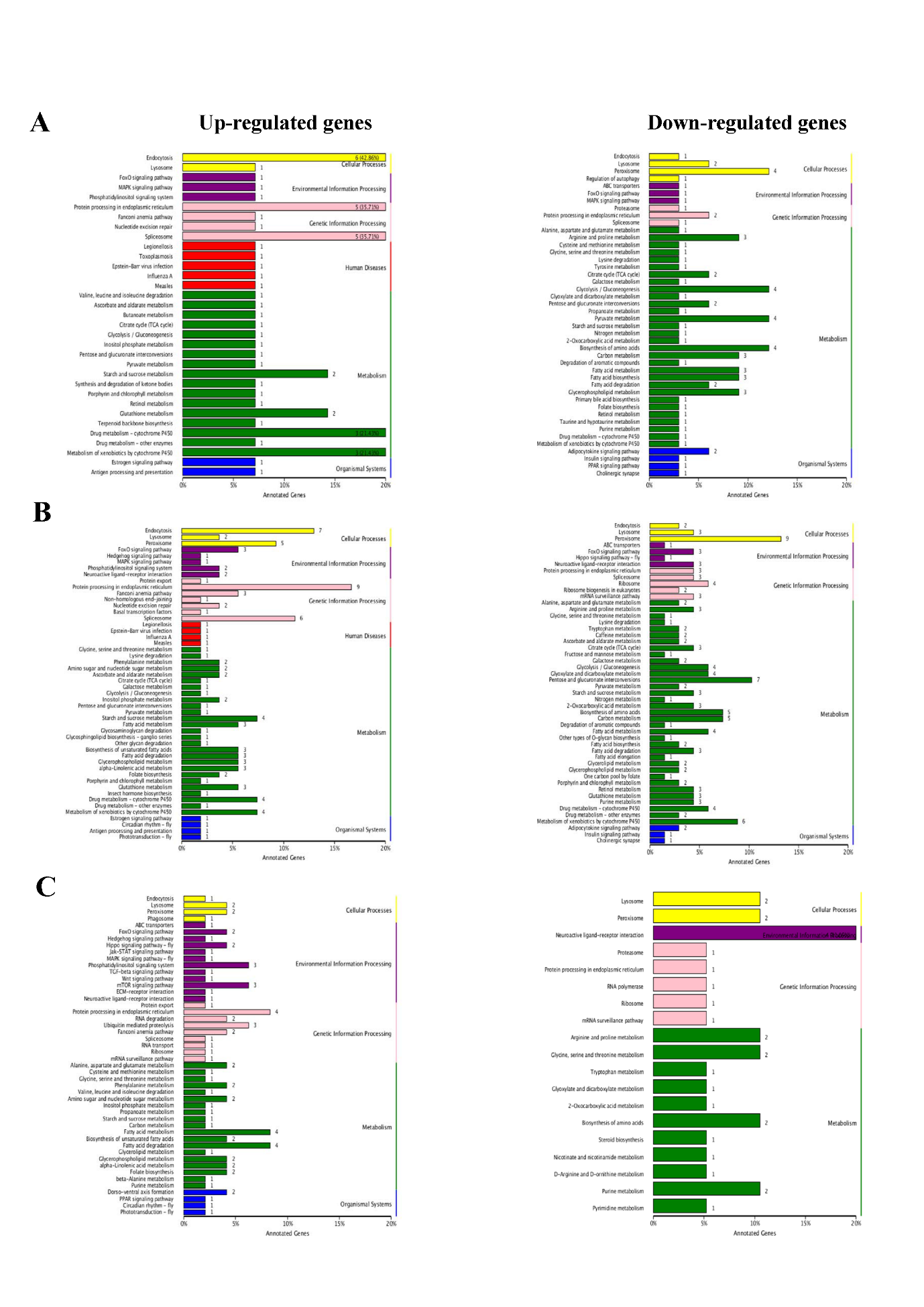

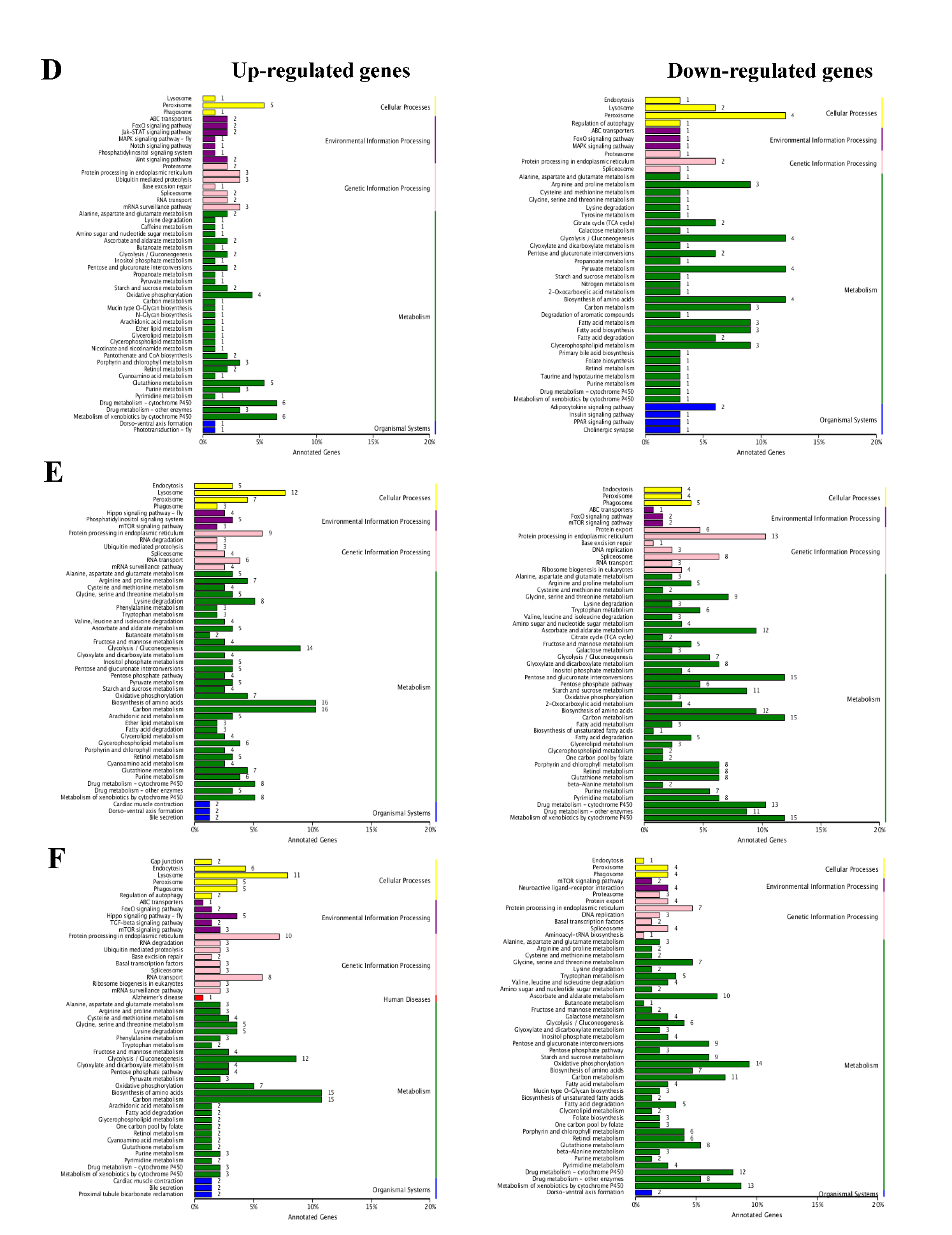
*

**S4. KEGG analysis of DEGs (up- and down-regulated genes) with fold change more than 2 in *B. correcta* and *B. dorsalis*.** A. 35 vs 25°C for *B. correcta.* B. 38 vs 25°C for *B. correcta.* C. 38 vs 35°C for *B. correcta.* D. 35 vs 25°C for *B. dorsalis.* E. 38 vs 25°C for *B. dorsalis.* F. 38 vs 35°C for *B. dorsalis.*


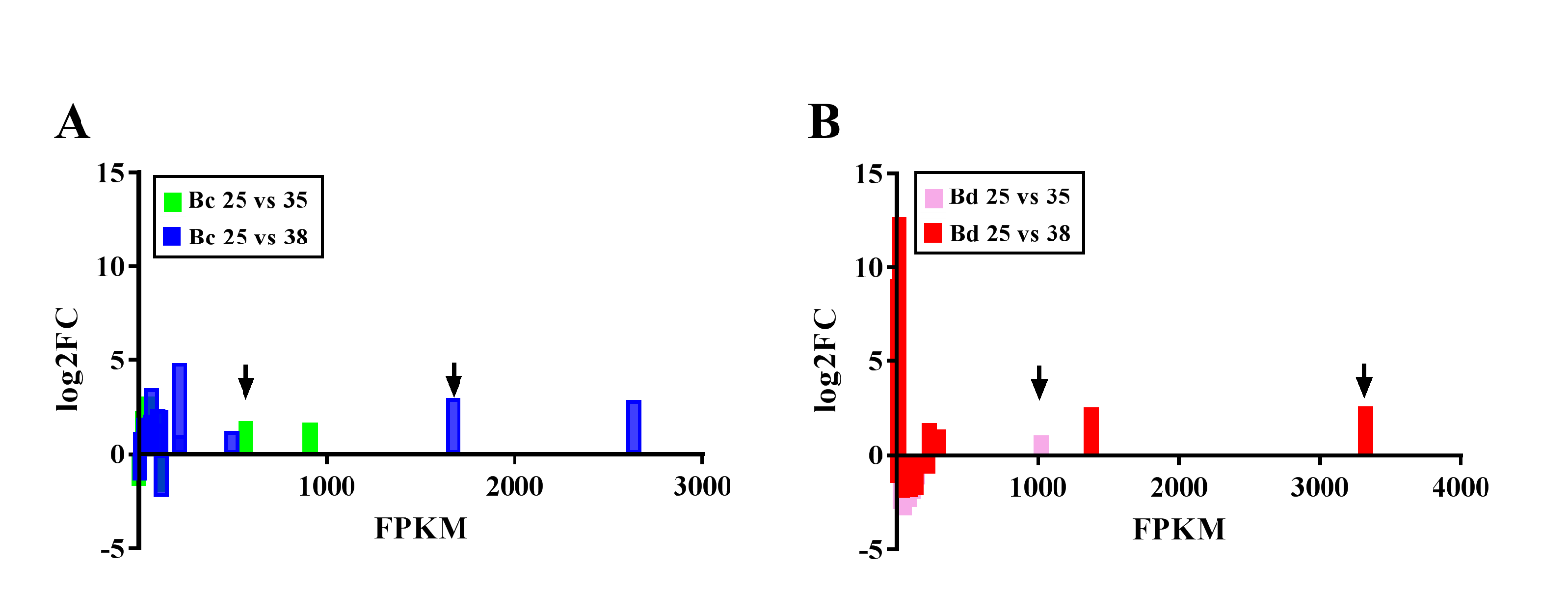


**S5. Comparisons of differentially expressed *Hsp* genes in response to thermal stress at two hardening temperatures in *B. correcta* and *B. dorsalis***. The bar charts indicate the expression level and fold change of differentially expressed *Hsp* genes in *B. correcta* and *B. dorsalis* at hardening temperatures of 35°C (green and pink bars) and 38°C (blue and red bars). Detailed data for transcript expression changes and related annotation information are reported in Table S7. Fold changes at each hardening temperature are expressed relative to 25°C, which served as the reference condition. The gene *Hsp23* is marked by a black arrow.

*
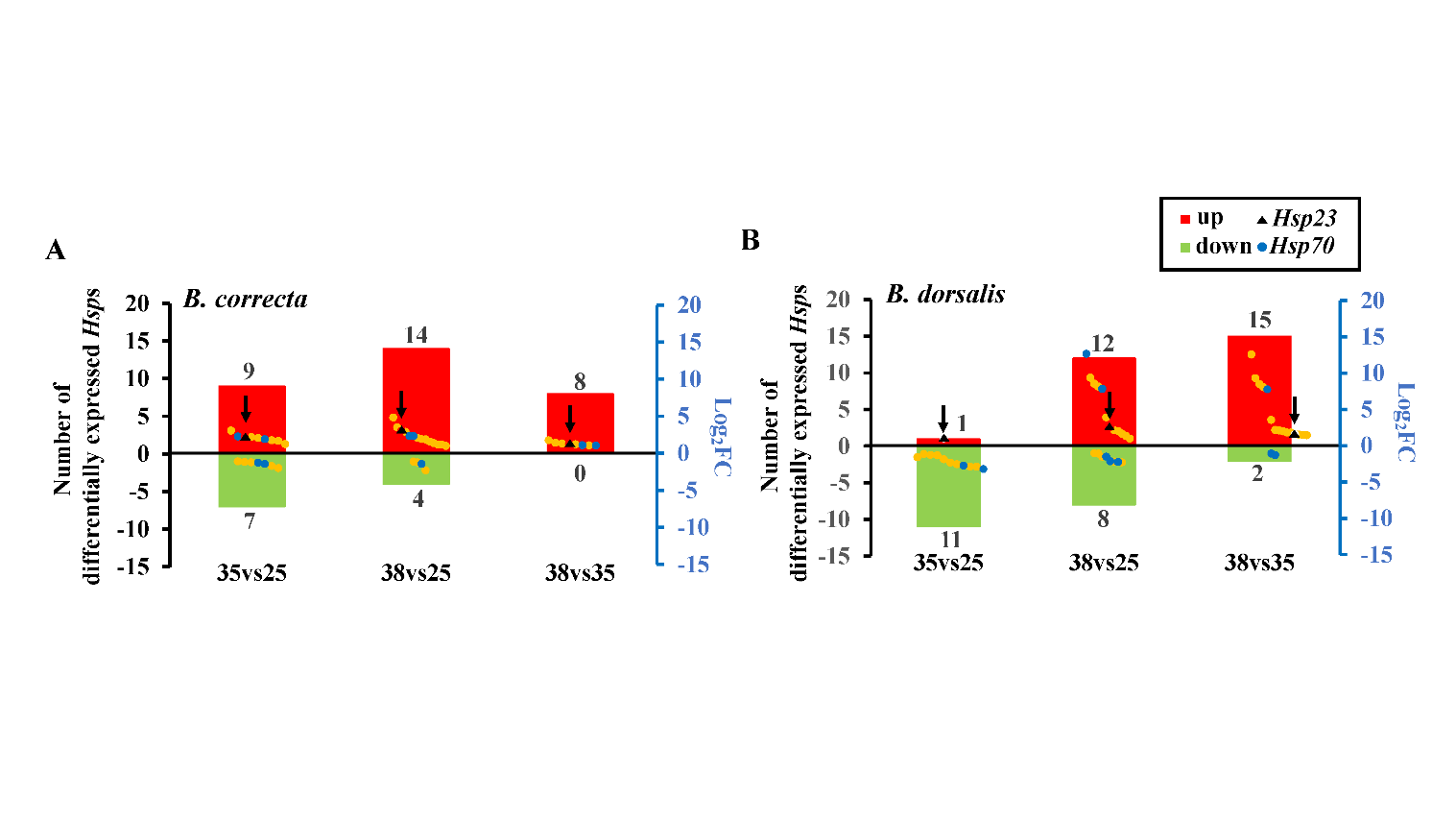
*

**S6. Numbers and relative fold change of differentially expressed *Hsp* genes in response to thermal stress at two hardening temperatures in *B. correcta* and *B. dorsalis***. Black arrows and triangles marked for *Hsp23* and blue dots marked for *Hsp70* highlight where they are positioned.

**

**

**S7. Weight of 3d, 6d, 9d-pupae after dsRNA-feeding in *B. dorsalis*.** The letter “*” above the bar represents a significant difference at P < 0.05, as determined by a t test. The error bars indicate 1SE**.**


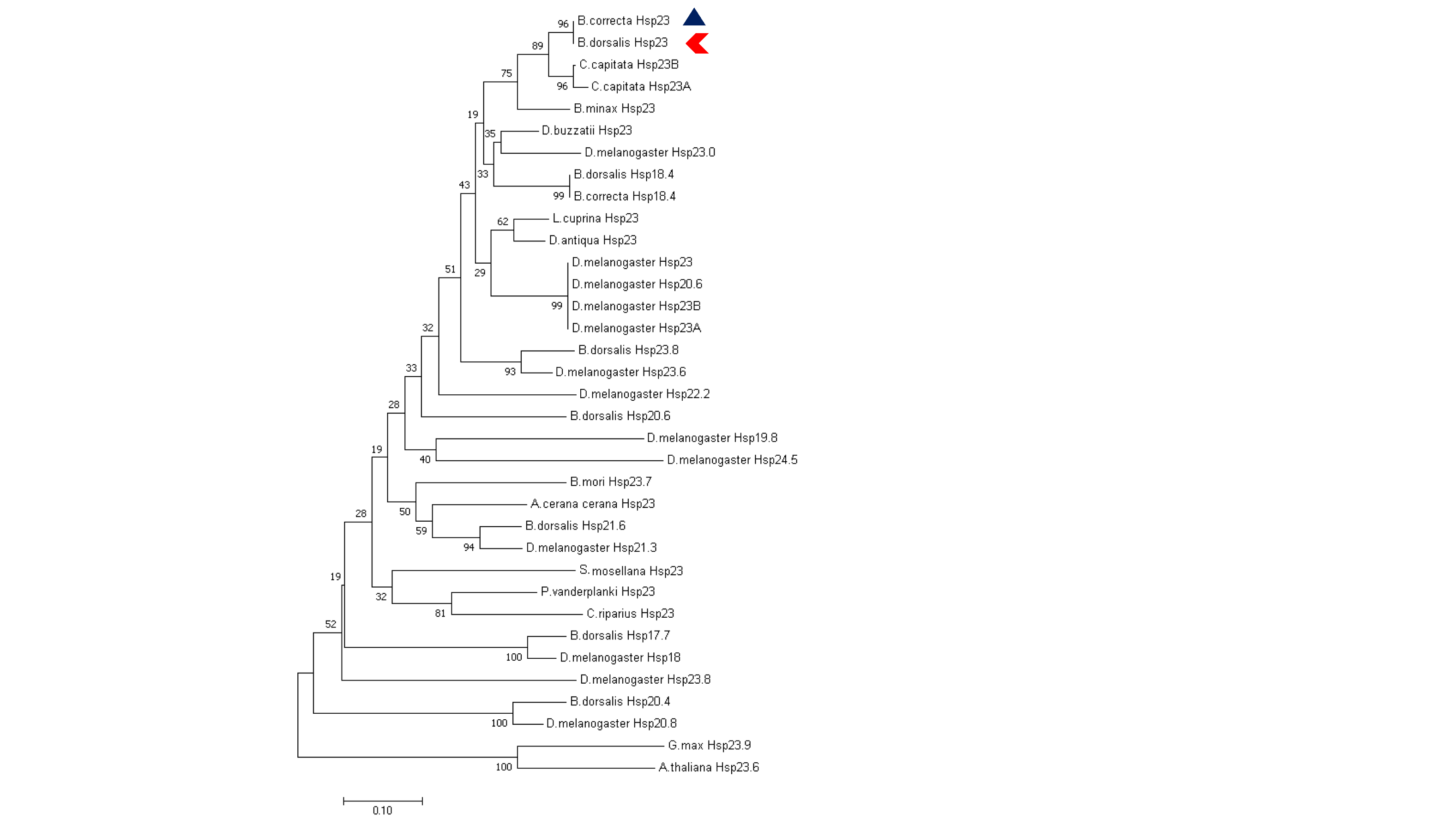


**S8. Phylogenetic analysis of s*Hsp* from different insect species.** The *B. correcta Hsp23* genes are labeled with a blue triangle and the *Hsp23* gene in *B. dorsalis* is labeled with a red arrow. The full names of the species and the accession numbers of the genes are designated with the corresponding abbreviations according to the supplementary information of Dou et al (2017)**.**

**Table S1. Statistics for larval unigenes assessment of the two *Bactrocera* species.**

| **Length Range** | **All Unigenes** | ***B. correcta* Unigenes** | ***B. dorsalis* Unigenes** |
| --- | --- | --- | --- |
| 200-300 | 43,063(45.42%) | 21,996(40.36%) | 26,778(47.51%) |
| 300-500 | 21,407(22.58%) | 12,820(23.52%) | 12,618(22.39%) |
| 500-1000 | 14,236(15.01%) | 8,817(16.18%) | 8,272(14.68%) |
| 1000-2000 | 8,372(8.83%) | 5,518(10.12%) | 4,862(8.63%) |
| 2000+ | 7,742(8.16%) | 5,352(9.82%) | 3,831(6.80%) |
| Total Number | 94,821 | 54,503 | 56,362 |
| Total Length | 67,215,743 | 43,182,339 | 35,832,653 |
| N50 Length | 1,409 | 1,658 | 1,132 |
| Mean Length | 708.87 | 792.29 | 635.76 |

**Table S2. Summary of gene annotation in the larval transcriptome.**

| **Anno_Database** | **Annotated Number** | **300<=length<1000** | **length>=1000** |
| --- | --- | --- | --- |
| NR_Annotation | 34,404 | 12,021 | 13,639 |
| Swiss-Prot_Annotation | 17,196 | 5,246 | 8,805 |
| GO_Annotation | 18,590 | 5,453 | 9,344 |
| COG_Annotation | 14,057 | 4,356 | 4,992 |
| KOG_Annotation | 22,784 | 7,429 | 9,843 |
| KEGG_Annotation | 12,767 | 4,054 | 6,279 |
| eggNOG4.5_Annotation | 35,656 | 12,048 | 12,896 |
| Pfam_Annotation | 25,011 | 8,017 | 11,078 |
| All_ | 42,931 | 15,244 | 13,798 |

**Table S3. Mapped ratios of each sample tested.**

| **ID** | **Clean Reads** | **Mapped Reads** | **Mapped Ratio** |
| --- | --- | --- | --- |
| Bc_35_1 | 24,467,975 | 19,015,084 | 77.71% |
| Bc_35_2 | 26,517,577 | 20,362,052 | 76.78% |
| Bc_35_3 | 25,889,946 | 20,391,174 | 78.76% |
| Bc_38_1 | 26,049,810 | 20,281,015 | 77.85% |
| Bc_38_2 | 30,392,565 | 23,404,345 | 77% |
| Bc_38_3 | 28,094,241 | 21,518,490 | 76.59% |
| Bc_25_1 | 27,672,942 | 21,463,337 | 77.56% |
| Bc_25_2 | 32,051,860 | 25,052,701 | 78.16% |
| Bc_25_3 | 24,306,536 | 18,990,774 | 78.13% |
| Bd_35_1 | 29,527,944 | 22,966,660 | 77.77% |
| Bd_35_2 | 30,089,439 | 24,176,757 | 80.34% |
| Bd_35_3 | 26,055,388 | 21,370,564 | 82.01% |
| Bd_38_1 | 22,628,457 | 18,204,932 | 80.45% |
| Bd_38_2 | 21,882,197 | 17,620,670 | 80.52% |
| Bd_38_3 | 20,701,365 | 16,801,917 | 81.16% |
| Bd_25_1 | 32,138,237 | 25,636,889 | 79.77% |
| Bd_25_2 | 34,113,202 | 26,810,347 | 78.59% |
| Bd_25_3 | 33,369,790 | 27,115,898 | 81.25% |

| **Primer** | **Sequence** | | **Size (bp)** |
| --- | --- | --- | --- |
| *Hsp23*-dsRNA-F | | ATGGCAAACCTACCATTGAT | 20 |
| *Hsp*23-dsRNA-R | | TTAAGCGCTTGCCTGCTCCT | 20 |
| *Hsp23*-F-T7 | | TAATACGACTCACTATAGGATGGCAAACCTACCATTGAT | 39 |
| *Hsp23*-R-T7 | | TAATACGACTCACTATAGGTTAAGCGCTTGCCTGCTCCT | 39 |
| *Hsp23*-dsRNA-qRT-F | | AACAAGCGCCCATCAAA | 17 |
| *Hsp23*-dsRNA-qRT-R | | AGAAGGCCGGTTCGTAG | 17 |
| *Hsp23*-trans-qRT-F | | ACACTTTGTTCGCCGCTAT | 19 |
| *Hsp23*-trans-qRT-R | | GCTTGCCTGCTCCTCAC | 17 |
| *Bc18srRNA*-qRT-F | | ACCAGGTCCGAACTTAAGCG | 20 |
| *Bc18srRNA*-qRT-R | | AACCAGACAAATCACTCCACGA | 22 |
| *BcHsp70*-qRT-F | | AGGAGTTGAGTTCAGGAA | 18 |
| *BcHsp70*-qRT-R | | TTATTGCGTTGTCGTTCA | 18 |
| *BcHsp90*-qRT-F | | GCTGACTTCTTGCGTTAT | 18 |
| *BcHsp90*-qRT-R | | GAGTTGCTTACTTGTTCCT | 19 |
| *Bd18srRNA*-qRT-F | | GCGAGAGGTGAAATTCTTGG | 20 |
| *Bd18srRNA*-qRT-R | | CGGGTAAGCGACTGAGAGAG | 20 |
| *BdHsp70*-qRT-F | | ACCAGCATACTTCAATGATT | 20 |
| *BdHsp70*-qRT-R | | TTCGTTAATGATTCGTAGCA | 20 |
| *BdHsp90*-qRT-F | | CCCAGTTCGGTTGGTCAG | 18 |
| *BdHsp90*-qRT-R | | TCGTTCTTGTCGGCTTCA | 18 |

**Table S4. Primers used in experiments.**

**Table S5. PCR efficiency of primers for genes in *B. dorsalis* and *B. correcta.***

| **Primer** | **Efficiency (%)** | **R^2^** |
| --- | --- | --- |
| *BdHsp23*-dsRNA-qRT | 105.306 | 0.997 |
| *BcHsp23*-dsRNA-qRT | 107.740 | 0.994 |
| *BdHsp23*-trans-qRT | 104.637 | 0.995 |
| *BcHsp23*-trans-qRT | 108.060 | 0.992 |
| *Bd18srRNA*-qRT | 100.001 | 1.000 |
| *Bc18srRNA*-qRT | 104.161 | 0.999 |
| *BdHsp70*-qRT | 108.195 | 0.997 |
| *BcHsp70*-qRT | 105.477 | 0.990 |
| *BdHsp90*-qRT | 106.694 | 0.999 |
| *BcHsp90*-qRT | 103.608 | 0.985 |



**Table S6. Expression stability of the candidate reference genes under different temperatures.**

The primers for reference genes were described by Shen et al (2013) and Hu et al (2014).

**Table S7. *Hsp* fold changes (FC) and annotation in DEGs.**

| **ID** | **log2FC** | **GO_ functional annotation** | **Annotation** |
| --- | --- | --- | --- |
| **Bc 35 vs 25℃** |  |  |  |
| Unigene_02827 | 3.07 | -- | *Hsp18.4*/PRE: *Hsp23* |
| Unigene_16574 | 2.26 | **response to stress**; ATP binding; | *Hsp70* |
| Unigene_28559 | 2.25 | **response to stress**; ATP binding; | *Hsp68* |
| Unigene_27981 | 2.21 | **response to stress**; ATP binding; | *Hsp70* |
| Unigene_65096 | 2.05 | **response to stress**; ATP binding; | PRE: *Hsp70* |
| Unigene_23303 | 1.90 | ATP binding; response to hypoxia; microtubule associated complex; heat shock-mediated polytene chromosome puffing | *Hsp70* |
| Unigene_62390 | 1.75 | **response to stress**; | *Hsp23* |
| Unigene_26609 | 1.67 | **response to stress**; | *Hsp20* |
| Unigene_25011 | 1.24 | **response to heat**; protein binding and refolding; microtubule associated complex; behavioral response to starvation; defense response to bacterium and fungus… | PRE: *Hsp27* |
| Unigene_05515 | -1.08 | -- | *Hsp20* |
| Unigene_00668 | -1.16 | -- | *Hsp20* |
| Unigene_03524 | -1.22 | -- | *Hsp67B2* |
| Unigene_10512 | -1.28 | **response to stress**; ATP binding; | *Hsp70* |
| Unigene_22343 | -1.44 | **response to stress**; ATP binding; | *Hsp70* |
| Unigene_03425 | -1.70 | -- | *Hsp100* |
| Unigene_01250 | -2.00 | ATP binding; polytene chromosome interband; lipid particle; protein folding; actin filament organization … | *Hsp83* |
| **Bc 38 vs 25℃** |  |  |  |
| Unigene_02827 | 4.83 | -- | *Hsp18.4*/PRE: *Hsp23* |
| Unigene_60561 | 3.51 | **response to stress**; | PRE: *Hsp23* |
| Unigene_62390 | 3.00 | **response to stress**; | *Hsp23* |
| Unigene_26609 | 2.89 | **response to stress**; | *Hsp20* |
| Unigene_23303 | 2.33 | ATP binding; response to hypoxia; microtubule associated complex; heat shock-mediated polytene chromosome puffing | *Hsp70* |
| Unigene_27981 | 2.32 | **response to stress**; ATP binding; | *Hsp70* |
| Unigene_28559 | 2.07 | **response to stress**; ATP binding; | PRE: *Hsp68* |
| Unigene_16574 | 1.91 | **response to stress**; ATP binding; | *Hsc70* |
| Unigene_65096 | 1.88 | **response to stress**; ATP binding; | PRE: *Hsp68* |
| Unigene_25011 | 1.59 | **response to heat**; protein binding and refolding; microtubule associated complex; behavioral response to starvation; defense response to bacterium and fungus… | PRE: *Hsp27* |
| Unigene_59390 | 1.38 | **response to heat**; behavioral response to starvation; defense response to bacterium and fungus; protein binding and refolding; determination of adult lifespan; | *Hsp20* |
| Unigene_09691 | 1.20 | **response to heat**; ATPase activity; porphobilinogen synthase activity; ATP binding; microtubule associated complex… | PRE: *Hsc70* |
| Unigene_00743 | 1.17 | **response to heat;** protein lipidation; regulation of autophagy; regulation of translational initiation by eIF2 alpha phosphorylation; response to methotrexate; | *Hsp67B3* |
| Unigene_00611 | 1.00 | **response to heat**; protein lipidation; embryo development; regulation of autophagy; regulation of translational initiation by eIF2 alpha phosphorylation … | *Hsp20* |
| Unigene_03425 | -1.12 | -- | *Hsp100* |
| Unigene_00668 | -1.24 | -- | *Hsp20* |
| Unigene_00850 | -1.45 | -- | *Hsp70* |
| Unigene_01250 | -2.28 | **response to heat; cold acclimation**; ATPase activity, coupled; ATP binding; protein folding and unfolded protein binding… | PRE: *Hsp83* |
| **Bc 38 vs 35℃** |  |  |  |
| Unigene_02827 | 1.76 | -- | *Hsp18.4*/PRE: *Hsp23* |
| Unigene_60561 | 1.41 | **response to stress**; | PRE: *Hsp23* |
| Unigene_00611 | 1.32 | **response to heat**; protein lipidation; embryo development; regulation of autophagy; regulation of translational initiation by eIF2 alpha phosphorylation … | *Hsp20* |
| Unigene_62390 | 1.25 | **response to stress**; | *Hsp23* |
| Unigene_26609 | 1.23 | **response to stress**; | *Hsp20* |
| Unigene_04370 | 1.09 | **response to heat**; porphobilinogen synthase activity; ATP binding; ATPase activity; porphyrin-containing compound biosynthetic process… | *Hsp70* |
| Unigene_59390 | 1.07 | **response to heat**; behavioral response to starvation; defense response to bacterium and fungus; protein binding and refolding; determination of adult lifespan; | *Hsp20* |
| Unigene_22343 | 1.03 | **response to stress**; ATP binding; | *Hsp70* |
|  |  |  |  |
| **Bd 35 vs 25℃** |  |  |  |
| Unigene_62390 | 1.03 | **response to stress**; | *Hsp23* |
| Unigene_09691 | -1.56 | **response to heat**; ATPase activity; porphobilinogen synthase activity; ATP binding; microtubule associated complex… | PRE: *Hsc70* |
| Unigene_00743 | -1.11 | **response to heat;** protein lipidation; regulation of autophagy; regulation of translational initiation by eIF2 alpha phosphorylation; response to methotrexate; | PRE: *Hsp67B3* |
| Unigene_25011 | -1.23 | **response to heat**; protein binding and refolding; microtubule associated complex; behavioral response to starvation; defense response to bacterium and fungus… | *Hsp27* |
| Unigene_59390 | -1.29 | **response to heat**; behavioral response to starvation; defense response to bacterium and fungus; protein binding and refolding; determination of adult lifespan; | *Hsp20* |
| Unigene_01250 | -1.83 | **response to heat; cold acclimation**; ATPase activity, coupled; ATP binding; protein folding and unfolded protein binding… | PRE: *Hsp83* |
| Unigene_61710 | -2.29 | **response to stress**; | PRE: *Hsp23* |
| Unigene_28559 | -2.52 | **response to stress**; ATP binding; | PRE: *Hsp68* |
| Unigene_27981 | -2.74 | **response to stress**; ATP binding; | *Hsp70* |
| Unigene_65096 | -2.84 | **response to stress**; ATP binding; | PRE: *Hsp68* |
| Unigene_60561 | -2.87 | **response to stress**; | PRE: *Hsp23* |
| Unigene_23303 | -3.20 | ATP binding; response to hypoxia; microtubule associated complex; heat shock-mediated polytene chromosome puffing | *Hsp70* |
|  |  |  |  |
| **Bd 38 vs 25℃** |  |  |  |
| Unigene_62365 | 12.66 | **response to stress**; ATP binding; 2-alkenal reductase [NAD(P)] activity; oxidation-reduction process; | *Hsc70* |
| Unigene_61339 | 9.40 | **response to stress**; ATP binding; protein folding and unfolded protein binding; | *Hsp90* |
| Unigene_62749 | 8.57 | -- | *Hsp20* |
| Unigene_57187 | 8.15 | cellular protein metabolic process; | *Hsp60* |
| Unigene_66085 | 7.85 | **response to heat**; determination of adult lifespan; ATP binding and ATP catabolic process; protein binding; reproduction… | *Hsp70* |
| Unigene_53284 | 3.92 | -- | *Hsp100* |
| Unigene_62390 | 2.57 | **response to stress**; | *Hsp23* |
| Unigene_65745 | 2.16 | -- | *Hsp90* |
| Unigene_65736 | 2.04 | -- | *Hsc70* |
| Unigene_53760 | 1.69 | **response to stress**; | PRE: *Hsp23* |
| Unigene_02827 | 1.37 | -- | Hsp18.4/PRE: *Hsp23* |
| Unigene_06111 | 1.01 | -- | *Hsp67B1* |
| Unigene_09691 | -1.01 | **response to heat**; ATPase activity; porphobilinogen synthase activity; ATP binding; microtubule associated complex… | PRE: *Hsc70* |
| Unigene_60561 | -1.06 | **response to stress**; | PRE: *Hsp23* |
| Unigene_57420 | -1.48 | -- | *Hsp70* |
| Unigene_01250 | -1.51 | **response to heat; cold acclimation**; ATPase activity, coupled; ATP binding; protein folding and unfolded protein binding… | PRE: *Hsc83* |
| Unigene_27981 | -2.13 | **response to stress**; ATP binding; | *Hsp70* |
| Unigene_65096 | -2.18 | **response to stress**; ATP binding; | PRE: *Hsp68* |
| Unigene_23303 | -2.24 | ATP binding; response to hypoxia; microtubule associated complex; heat shock-mediated polytene chromosome puffing | *Hsp70* |
| Unigene_28559 | -2.26 | **response to stress**; ATP binding; | PRE: *Hsp68* |
|  |  |  |  |
| **Bd 38 vs 35℃** |  |  |  |
| Unigene_62365 | 12.59 | **response to stress**; ATP binding; 2-alkenal reductase [NAD(P)] activity; oxidation-reduction process; | *Hsc70* |
| Unigene_61339 | 9.33 | **response to stress**; ATP binding; protein folding and unfolded protein binding; | *Hsp90* |
| Unigene_62749 | 8.51 | -- | *Hsp20* |
| Unigene_57187 | 8.08 | cellular protein metabolic process; | *Hsp60* |
| Unigene_66085 | 7.78 | **response to heat**; determination of adult lifespan; ATP binding and ATP catabolic process; protein binding; reproduction… | *Hsp70* |
| Unigene_53284 | 3.56 | -- | *Hsp100* |
| Unigene_53760 | 2.20 | **response to stress**; | PRE: *Hsp23* |
| Unigene_02827 | 2.12 | -- | *Hsp18.4*/PRE: *Hsp23* |
| Unigene_06111 | 2.01 | -- | *Hsp67B1* |
| Unigene_60561 | 1.85 | **response to stress**; | PRE: *Hsp23* |
| Unigene_65745 | 1.85 | -- | *Hsp90* |
| Unigene_62390 | 1.57 | **response to stress**; | Hsp23 |
| Unigene_26609 | 1.56 | **response to stress**; | *Hsp20* |
| Unigene_00743 | 1.50 | **response to heat;** protein lipidation; regulation of autophagy; regulation of translational initiation by eIF2 alpha phosphorylation; response to methotrexate; | PRE: *Hsp67B3* |
| Unigene_65736 | 1.46 | -- | *Hsc70* |
| Unigene_90903 | -1.07 | -- | *Hsp70* |
| Unigene_26405 | -1.29 | ATP binding; extracellular space; lipid particle; protein folding; multicellular organism reproduction; | PRE: *Hsp70* |

“PRE” represents “predict”.
